# Functionally related internal fluctuations in human ileal bile acid‐binding protein by high pressure nuclear magnetic resonance

**DOI:** 10.1002/pro.70608

**Published:** 2026-05-04

**Authors:** Tamara Teski, Bence Balterer, Gergő Horváth, Gábor Turczel, Orsolya Toke

**Affiliations:** ^1^ Centre for Structural Science HUN‐REN Research Centre for Natural Sciences Budapest Hungary; ^2^ Doctoral School of Biology, Institute of Biology ELTE Eötvös Loránd University Budapest Hungary

**Keywords:** bile salts, conformational exchange, high pressure NMR, intracellular lipid‐binding proteins, invisible states, ligand binding, order–disorder transitions, pressure denaturation, protein dynamics, protein folding

## Abstract

Hidden protein states with partially unfolded regions can have a functional relevance and may also contribute to an uncontrolled self‐association of proteins leading to pathological conditions. Human ileal bile acid‐binding protein (hI‐BABP), a member of the family of intracellular lipid‐binding proteins (iLBPs), plays a key role in the transcellular trafficking and metabolic targeting of bile salts. Disorder–order transitions and sparsely populated hidden states in hI‐BABP are thought to be key elements of ligand recognition, with implications for bile salt recycling and bile salt‐mediated signaling events. To improve our understanding of the structural determinants of hI‐BABP stability, high pressure NMR was used to probe local packing interactions and characterize the folding/unfolding process. Heterogeneity in pressure response, revealed by residue‐specific analysis of chemical shift and intensity changes, indicates a deviation from two‐state unfolding. Four specific protein regions such as the N‐terminal β‐strand, the helical cap, a hydrophobic cluster at the bottom of the β‐barrel, and segments of the HIJ‐region have been found to exhibit a pressure response differing significantly from the rest of the protein. Our analysis further shows that a low‐populated higher‐energy state, inferred by NMR relaxation dispersion measurements, becomes less distinct from the ground state at high pressure, corroborating the hypothesis that conformational exchange between a closed and a more open EFGH‐region, which mediates ligand entry, is related to a partial unfolding of the protein. A pathway of destabilization induced by high hydrostatic pressure is proposed and discussed in relation to the response of iLBPs to other stress conditions.

## 1 INTRODUCTION

Human ileal bile acid‐binding protein (hI‐BABP) is a member of the family of intracellular lipid‐binding proteins (iLBPs), a class of small (14–16 kDa), predominantly β‐sheet proteins that are thought to facilitate the cellular trafficking of fatty acids, retinoids, and bile salts (Banaszak et al., 1994; Veerkamp & Maatman, 1995). Human I‐BABP is abundantly expressed in the enterocytes of the distal small intestine and plays a key role in the enterohepatic circulation of bile salts (Borgström et al., 1985; Hofmann, 2009; Small et al., 1972). In addition, by controlling the presentation of bile salts to the farnesoid X receptor, BABPs also have a role in gene regulation and, thereby, in the control of lipid, glucose, and energy metabolism (Chiang & Ferrell, 2020; Houten et al., 2006; Makishima et al., 1999; Parks et al., 1999). Malfunctioning of BABPs has been related to a wide range of metabolic diseases (Ding et al., 2015; Lefebvre et al., 2009; Perino et al., 2021) and, more recently, to cancer development (Bintee et al., 2025).

**FIGURE 1:**
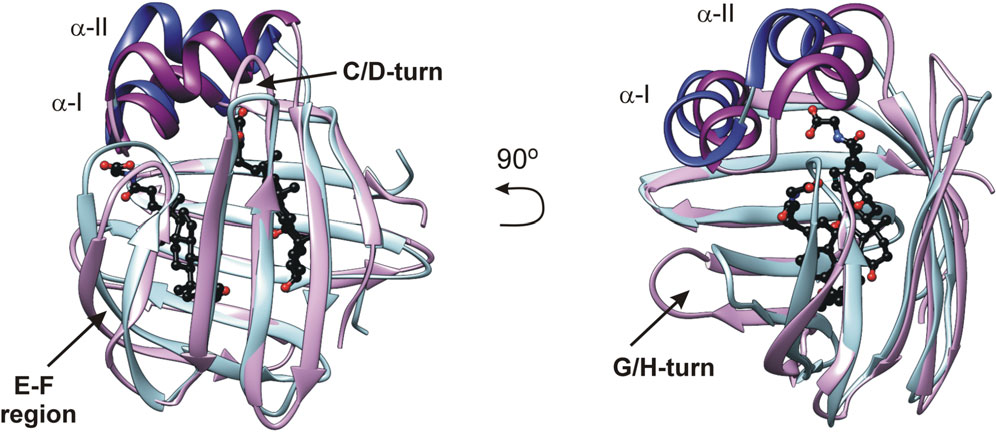
Superimposed ribbon diagrams of *apo* human I‐BABP (PDB: 1O1U; Kurz et al., 2003, blue) and the heterotypic doubly‐ligated complex of hI‐BABP with glycocholate and glycochenodeoxycholate (PDB: 2MM3; Horváth et al., 2016, pink). The most representative member of the lowest‐energy NMR structural ensemble is shown for both ligation states. Regions undergoing the most significant backbone conformational change upon ligand binding are highlighted.

Human I‐BABP has a conserved topology comprised of two orthologous five‐stranded antiparallel β‐sheets covered by a helix–loop–helix motif, enclosing a binding cavity of ~1000 Å (Figure 1) (Horváth et al., 2016; Kurz et al., 2003). Unlike other subfamilies of iLBPs, that is, fatty acid‐binding proteins (FABPs) and cellular retinal (retinoic acid)‐binding proteins (CR(A)BPs), which bind a single molecule of ligand (Banaszak et al., 1994), the internal cavity of BABPs accommodates two binding sites (Toke, 2022). The human ileal BABP analogue binds two molecules of glycocholate, the physiologically most abundant bile salt, with low intrinsic affinity but a high degree of positive cooperativity (Tochtrop et al., 2002). The energetic communication between the two binding sites has been shown to be governed by the hydroxylation pattern of the bound bile salts (Tochtrop et al., 2003; Toke et al., 2006). In addition to binding cooperativity, di‐ and trihydroxy bile salts display a high degree of site preference upon interacting with hI‐BABP, with the ability to displace each other from site 1 and site 2, respectively (Horváth et al., 2022; Teski et al., 2025; Tochtrop et al., 2004; Toke et al., 2006). The solution NMR structure of the thermodynamically most stable heterotypic complex of hI‐BABP with glycocholate and glycochenodeoxycholate reveals an extensive network of hydrogen bonds that, together with hydrophobic interactions, stabilize the bound bile salts (Horváth et al., 2016).

Disorder–order transitions and hidden protein states have a major role in iLBP‐ligand recognition (Ragona et al., 2014; Toke, 2022). In BABPs, NMR relaxation data provide evidence of a ms‐timescale conformational fluctuation in the *apo* state, which ceases upon bile salt binding (Cogliati et al., 2010; Horváth et al., 2012; Horváth et al., 2014; Ragona et al., 2006). In the human analogue, the timescale of the exchange matches that of a rate‐limiting conformational change preceding bile salt binding (Toke et al., 2007), indicating a direct link between global internal motions and the binding process. NMR spectroscopic measurements further show that the low‐populated (~1%–5%) higher energy state is reminiscent of a *holo*‐like conformation exhibiting a more open EFGH‐region (Horváth et al., 2016). Temperature‐dependent NMR relaxation measurements reveal an entropy‐enthalpy compensation in the thermodynamics of the exchange characteristic of disorder–order transitions (Horváth et al., 2014). The protonation equilibria of histidines are thought to provide a pH‐dependent regulation of a transition between a closed and a more open state mediating ligand entry in both the chicken liver (Eliseo et al., 2007; Ragona et al., 2006) and the human ileal BABP analogue (Horváth et al., 2016, 2019).

Beyond their role in regulating ligand binding, conformational fluctuations in BABPs and other iLBPs also have implications for transferring ligands between cell compartments. Specifically, within the framework of the “dynamic portal hypothesis” (Hodsdon & Cistola 1997a, 1997b), collision with the acceptor membrane has been proposed to shift an order–disorder equilibrium involving α‐II and the C/D‐turn of ligated FABPs toward a more disordered state, thereby facilitating ligand transfer (Corsico et al., 1998; Herr et al., 1995; Kim & Storch, 1992). As I‐BABPs from specific species have been found to be functionally associated and co‐localized with the ileal bile acid transporter in the vicinity of the plasma membrane (Kramer et al., 1993; Nakahara et al., 2005), disorder–order transitions induced by the membrane environment may have functional relevance for cytosolic bile acid transport in enterocytes. In addition to ligand transfer mechanisms, conformational fluctuations in BABPs may also play a role in nuclear translocation and bile acid signaling, specifically in the exposure of specific residues in a geometrical arrangement appropriate for signaling (Ayers et al., 2007; Sessler & Noy, 2005). Given the multiple roles of disorder–order transitions in BABP function, elucidation of the determinants of stability and flexibility in the protein family is of great importance for understanding their action in cellular processes.

As a single‐domain protein composed predominantly of antiparallel β‐strands with a small hydrophobic core, the folding mechanism of iLBPs has been a subject of interest (Budyak et al., 2013; Burns et al., 1998; Delassio & Ropson, 2000; D'Onofrio et al., 2009; Hodsdon & Frieden, 2001; Kim & Frieden, 1998; Li & Frieden, 2007a, 2007b; Ropson et al., 2009; Ropson & Frieden, 1992; Thakur et al., 2018). Although there are substantial differences in folding rates and the number and types of intermediate states across iLBP subfamilies, hydrophobic collapse is thought to be a conserved mechanism for the initiation of folding in the family. Site‐specific ^19^F NMR monitoring of aromatic residues during urea denaturation indicates the presence of native‐like hydrophobic contacts in the unfolded state of iLBPs, which may act as nucleation sites for folding (Basehore & Ropson, 2011; Li & Frieden, 2007a, 2007b; Ropson & Frieden, 1992). Following the initial burst phase, one or two slower kinetic steps have been attributed to β‐strand formation (Yeh et al., 2001) and side chain stabilization (Chattopadhyay et al., 2002). Intriguingly, despite their highly homologous structures, the locations of nucleation sites appear to differ between FABPs (Ropson et al., 2006) and BABPs (Basehore & Ropson, 2011), a matter that remains under debate. Moreover, while most kinetic studies in the presence of denaturants indicate a multistate folding/unfolding mechanism for iLBPs (Burns & Ropson, 2001; Li & Frieden, 2007a, 2007b; Ropson et al., 2009; Yeh et al., 2001), fluorescence and CD kinetic analyses of urea‐induced unfolding of hI‐BABP report a single phase (Ropson et al., 2009). The factors underlying these differences in the folding behavior of homologous iLBPs (even from the same tissue in different organisms) are not yet fully understood, but the lack of an appropriate reporter group may pose a challenge by hindering the detection of intermediate states.

While most folding studies of iLBPs have addressed chemical denaturation, here we focus on pressure‐induced processes (Akasaka, 2006; Schroer et al., 2010; Smeller, 2002). As unfolding by different stress conditions is dictated by different mechanisms, they provide complementary information on the process (Pastore & Temussi, 2022; Roumestand et al., 2025). Specifically, while chemical denaturants preferentially interact with the protein backbone, inducing uniform destabilization of the structure, high hydrostatic pressure promotes the hydration of solvent‐excluded cavities, selectively destabilizing hydrophobic interactions (Chen & Makhatadze, 2017; Roche et al., 2012). Pressure denaturation combined with NMR spectroscopy, enabling a residue‐level monitoring of the pressure response, has proven to be a powerful approach for elucidating packing interactions and detecting folding intermediates (Berner & Kovermann, 2024; Caro & Wand, 2018; Roche et al., 2017). In the present study, to improve our understanding of the determinants of hI‐BABP stability and functionally relevant flexibility, we investigated the pressure‐induced unfolding of the protein. To our knowledge, this represents the first high‐pressure analysis of the folding–unfolding transition in an iLBP. Our residue‐level analysis indicates a multistate unfolding mechanism for hI‐BABP, identifying four key regions that behave distinctly from the rest of the protein and may be involved in the formation of an intermediate. Chemical shift and NMR relaxation analyses further suggest a link between pressure‐induced conformational fluctuations in the pretransition range and functionally relevant conformational exchange in the native state. Comparison of slow dynamics at atmospheric and high pressure supports the hypothesis that a hidden conformer involved in the regulation of ligand entry is related to a partially unfolded state of hI‐BABP. A pathway of destabilization is proposed and discussed in the context of the folding/unfolding behavior of iLBPs under other stress conditions.

## 2 RESULTS

### 2.1 Structural information from pressure‐dependent 1D NMR spectra

**FIGURE 2:**
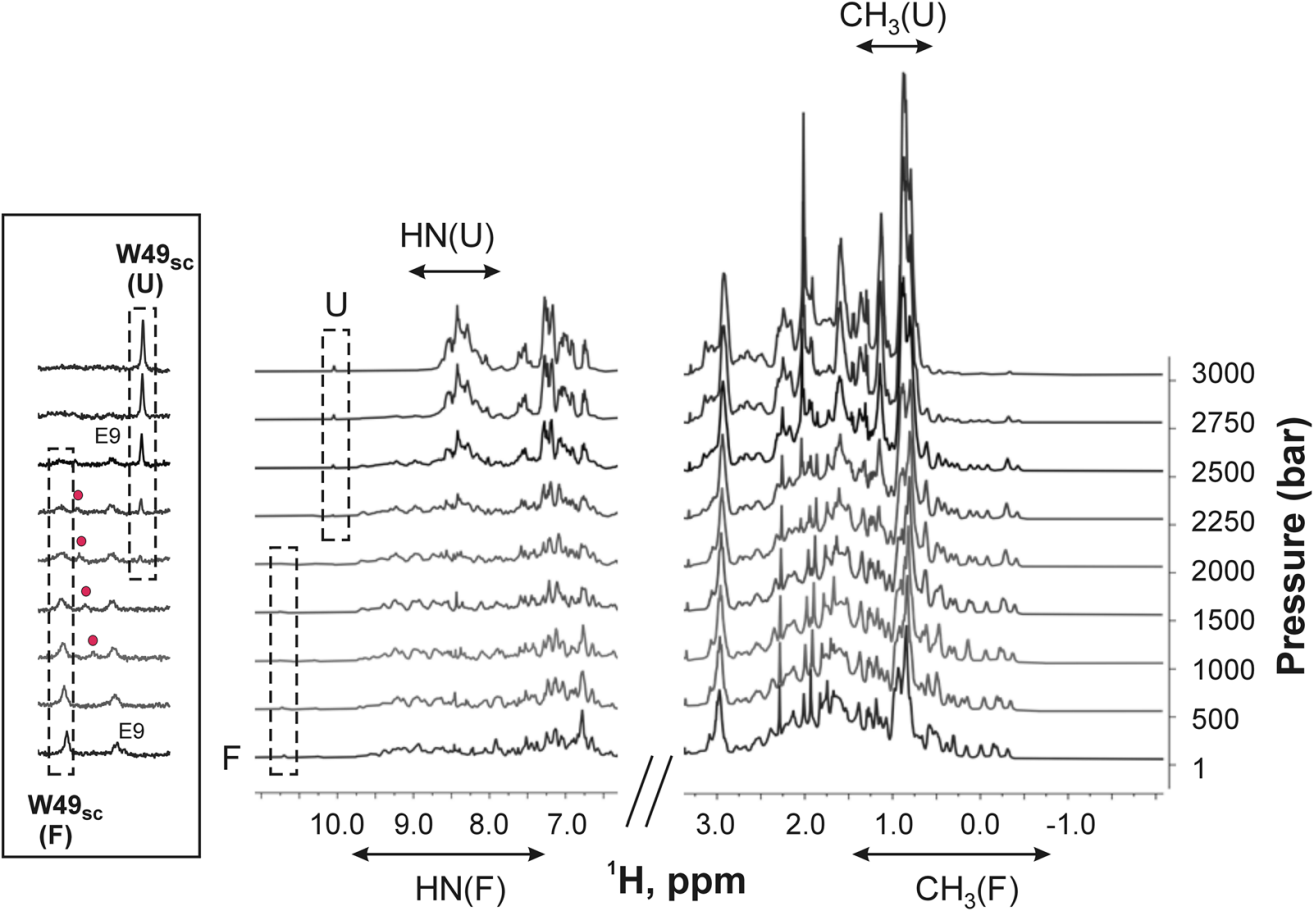
Stacked‐plot of one‐dimensional ^1^H‐NMR spectra of hI‐BABP (0.4 mM, pH = 6.3) recorded as a function of pressure (18°C, 600 MHz). Regions corresponding to the amide HN and the methyl resonances are highlighted. The decrease with pressure of the W49 side chain resonance corresponding to the folded state (10.72 ppm) with a concomitant appearance of a new peak at ~2000 bar representing the unfolded state (10.06 ppm) is shown in large in the left. Note the intermediate resonance between the folded and unfolded peaks of W49^sc^ (red dot). (The peak at 10.27 ppm corresponds to the backbone amide of E9.)

One‐dimensional ^1^H NMR spectra of hI‐BABP as a function of pressure show a characteristic loss of chemical shift dispersion in the amide NH and aromatic regions starting around 2000 bar, indicative of increasing disorder in the polypeptide chain (Figure 2). The side‐chain NH resonance of the buried single tryptophan (W49^sc^) undergoes a marked shift from 10.72 to 10.06 ppm, corresponding to the folded and unfolded states, respectively, consistent with previous observations using urea denaturation (Horváth et al., 2017). In addition, at 1000 bar, a new resonance appears at a chemical shift between the folded and unfolded peaks of the W49 side chain. The new peak reaches maximum intensity at ~2000 bar, where the folded form of W49^sc^ has lost more than 50% of its intensity, and the unfolded form begins to emerge.

Spectral changes in the amide NH and aromatic regions are paralleled by the intensity loss of side‐chain methyl proton signals in the −0.5 to 0.4 ppm spectral range arising from buried hydrophobic residues. Their peak intensity decreases substantially by 2500 bar and nearly disappears by 3000 bar, consistent with the loss of native packing interactions. Careful inspection of the upfield region of the spectra also reveals subtle changes among the methyl resonances in the 1–2000 bar range, indicating a perturbation of the protein structure in response to increasing pressure. This occurs in the pretransition range, where chemical shift dispersion is preserved and overlaps with the pressure range where the intermediate resonance (10.50 ppm, 1000 bar–10.63 ppm, 2500 bar) gains intensity. As indicated by chemical shifts and resonance intensities, spectral changes in the investigated pressure range of 1–3000 bar are fully reversible (Figures S1, S2).

### 2.2 Conformational fluctuations obtained from pressure‐dependent ^1^H‐ and ^15^N‐chemical shifts

**FIGURE 3:**
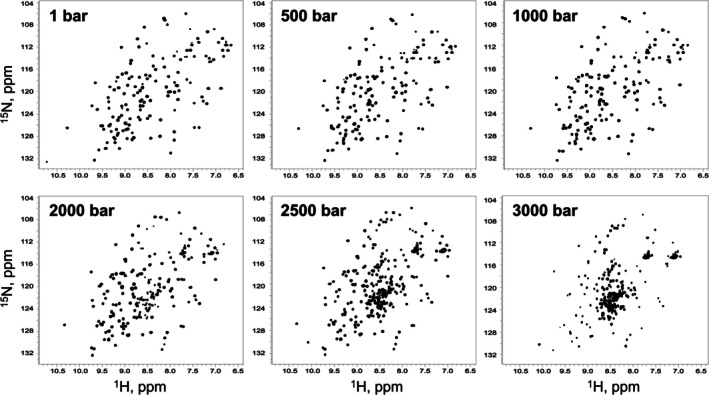
Pressure‐induced unfolding of hI‐BABP monitored by two‐dimensional NMR. Examples of ^1^H‐^15^N HSQC NMR spectra of ^15^N‐labeled hI‐BABP (18°C, pH = 6.3) at different pressures. Crowding in the middle of the spectrum, characteristic of disordered polypeptide chains, begins to appear at 1750–2000 bar.

To explore pressure‐induced conformational fluctuations in hI‐BABP at the residue level, two‐dimensional NMR experiments were performed under variable pressure. Figure 3 shows a series of ^1^H‐^15^N HSQC spectra at 18°C and pH 6.3 at specific hydrostatic pressures in the range of 1–3000 bar. As cross‐peaks exhibiting chemical shift dispersion shifted continuously with pressure, the assignment of resonances was carried out by transferring the assignment obtained previously at atmospheric pressure under the same experimental conditions (Horváth et al., 2014). Up to 1500 bar, backbone NH cross‐peaks remain well dispersed and retain high intensity, with no appearance of new peaks. In the 1500–2000 bar range, intensity loss becomes apparent for most resonances, whereas chemical shift dispersion is largely preserved. Around 2000 bar, new peaks begin to appear, and by ~2500 bar, significant crowding occurs in the central part of the spectrum, indicative of a disordered polypeptide chain. These observations are consistent with one‐dimensional ^1^H spectra, which show changes in the chemical environment of both the backbone and buried hydrophobic side chains in the corresponding pressure range. By 3000 bar, the intensity of dispersed peaks reflecting the folded conformer decreases to ~20%–30% of the original, accompanied by increased spectral crowding in the 8.3–8.7 ppm (^1^H), 121–125 ppm (^15^N) chemical shift range. This indicates the coexistence of at least two distinct populations of hI‐BABP in the 2000–3000 bar range: a population that retains a substantial native‐like structure undergoing conformational fluctuations, which are fast on the NMR timescale (τ < < ms), and a more disordered population en route to the completely unfolded state.

**FIGURE 4:**
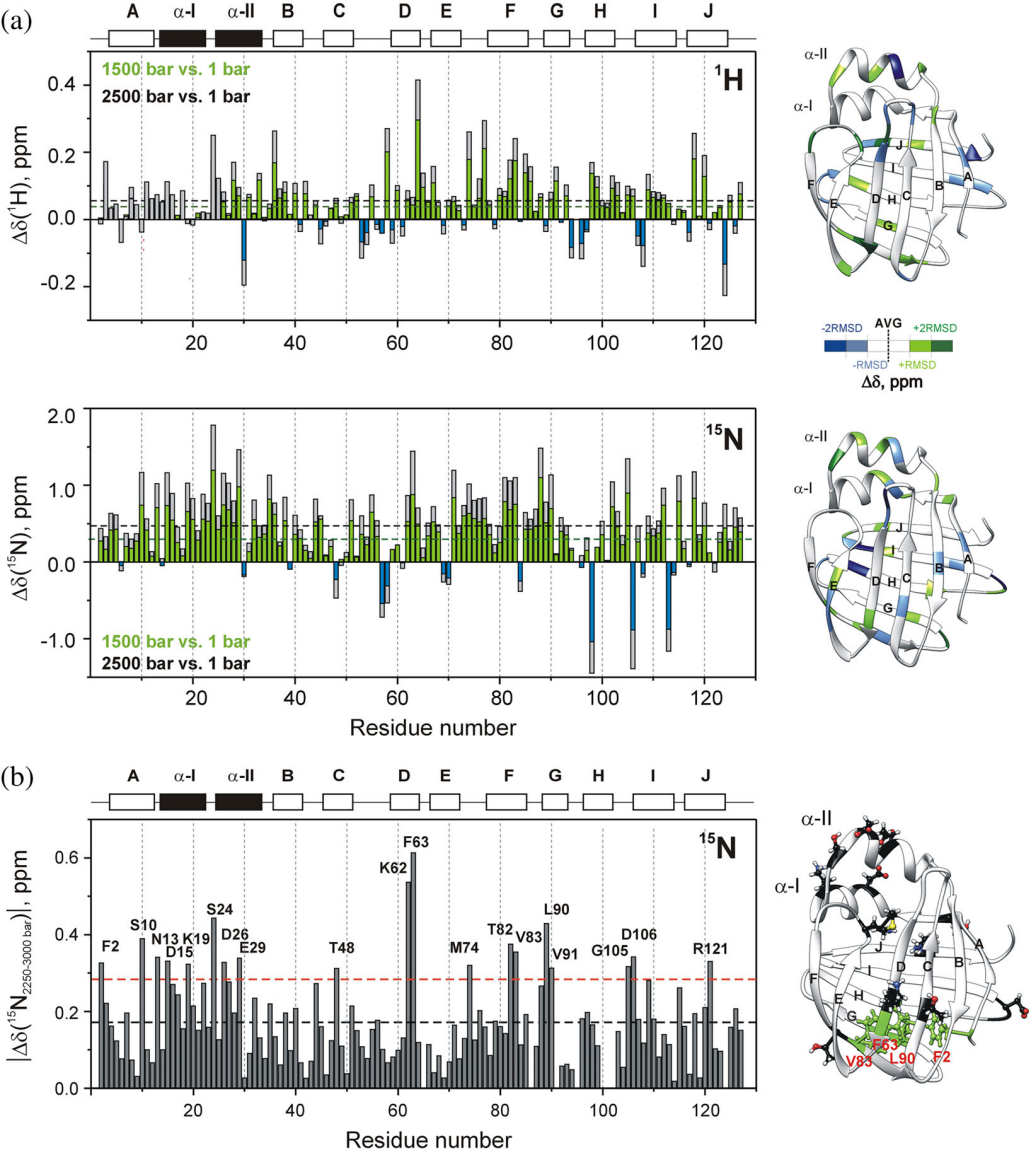
Pressure‐induced chemical shift changes in hI‐BABP (18°C, pH = 6.3). Pressure‐induced changes in (a) ^1^H (top) and ^15^N (bottom) chemical shifts of backbone NH resonances between 1 and 1500 bar (green, blue) and 1 and 2500 bar (gray) as a function of the amino acid sequence. Dotted lines correspond to the average values. Secondary structural elements are indicated at the top. Residues exhibiting the most significant chemical shift changes are as follows: For ^1^H, T58, T64, M74, K77, V83, T118 (>2 rmsd) and S24, I28, A33, I36, K67, T82, M85, Q86, V91, H98, E120 (>1 rmsd); for ^15^N, S24, G88 (>2 rmsd) and S10, N13, D15, D26, E29, K35, F63, I71, A81, L90, V83, E102, G105, S112, G115, T118 (>1 rmsd). Residues with deviation from the average by more than one standard deviation are mapped on the ribbon diagram of the protein (PDB: 1O1U) in a color coding depicted in the figure. (b) ^15^N chemical shift changes between 2250 and 3000 bar as a function of the amino acid sequence. Dotted lines correspond to the average value (black) and the average + rmsd (red). Secondary structural elements are indicated at the top. Residues with deviation from the average by more than one standard deviation are labeled and mapped on the ribbon diagram of the protein. A hydrophobic cluster at the bottom of the β‐barrel with outstanding ^15^N chemical shift changes is highlighted.

To gain insight into the early events of pressure‐induced conformational fluctuations, chemical shift changes were first analyzed over the 1–1500 bar range (Figure 4a). In the absence of new peaks, spectral changes in this pressure range reflect conformational fluctuations within the folded state. Specifically, according to the relation between compressibility and volume fluctuations in proteins (Cooper, 1976), chemical shift changes arising from local conformational alterations in response to pressure reflect part of the protein's microscopic volume fluctuation. With a few exceptions, pressure‐induced chemical shift changes in hI‐BABP were toward higher resonance frequencies, primarily arising from the shrinkage of amide NH‐carbonyl hydrogen bonds (shorter H · O distance causes a stronger polarization of the hydrogen bond resulting in less shielding of the magnetic field) accompanied by minor changes in secondary and tertiary structure. Between 1 and 1500 bar, chemical shift changes averaged at 0.04 ± 0.07 ppm (^1^H) and 0.34 ± 0.36 ppm (^15^N), comparable to values reported for BPTI (Li et al., 1998; Williamson et al., 2003), suggesting similar backbone hydrogen‐bond shortenings as well (0.03 ± 0.12 Å). Residues exhibiting significant ^1^H chemical shift changes were mainly located at the termini of secondary structural elements, with exceptions in α‐II and in a segment of βF (T82‐Q86), including a break that links one face of the protein to the other (Figure 4a, top). Regarding ^15^N chemical shifts, which in addition to hydrogen bonds, are sensitive to alterations in backbone torsional angles as well, changes were most prevalent in the helical region and at the bottom of the β‐barrel, affecting specific hydrophobic residues in the DEF β‐strands (Figure 4a, bottom). Significantly above average (>2 rmsd) negative changes for ^1^H and ^15^N chemical shifts were limited to K30 (α‐II), K124 (βJ) and H57 (C/D‐turn), H98 (βH), D106 (H/I‐turn), T113 (βI), respectively. Notably, the most significantly affected residues were part of a cluster undergoing slow conformational exchange in the native state (Horváth et al., 2012, 2014, 2016), suggesting a connection between ms‐timescale fluctuations in native hI‐BABP and pressure‐induced processes.

Above 1500 bar, chemical shifts continue to change throughout the polypeptide chain. However, when normalized to pressure increase, a slight decrease is observed for both ^1^H (0.029 ppb/bar vs. 0.040 ppb/bar) and ^15^N (0.220 ppb/bar vs. 0.276 ppb/bar). In the last 750 bar of the experimentally available pressure range (2250–3000 bar) (Figure 4b), ^15^N shift changes exceeding the average by more than one standard deviation are observed in the helical segment and at the bottom of the β‐barrel, including four hydrophobic residues (F2, F63, V83, L90) and their immediate vicinity. Despite being located in different β‐strands, these residues are part of a hydrophobic cluster in the native state. The fact that they experience above average changes in their chemical environment over a pressure range where a significant fraction of the protein is already disordered suggests a role in the final steps of unfolding and, given the fully reversible nature of the process, possibly in early folding events, such as hydrophobic collapse.

### 2.3 Multistate unfolding process indicated by pressure‐dependent ^1^H/ ^15^N cross‐peak intensities

**FIGURE 5:**
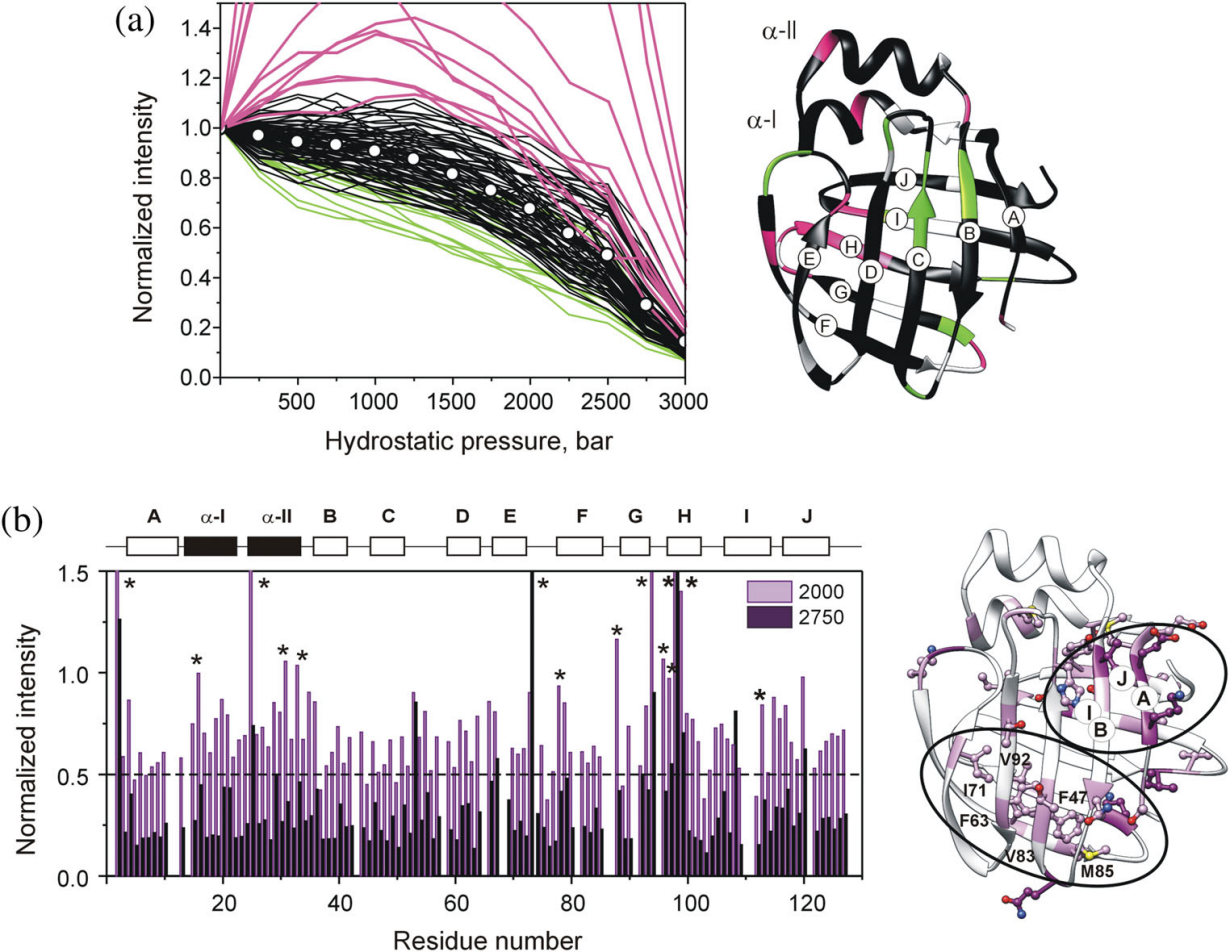
Pressure‐induced changes in resonance intensities. (a) Overlay of residue‐specific pressure‐induced denaturation curves obtained for hI‐BABP. Cross‐peak intensities of ^1^H‐^15^N HSQC NMR spectra have been normalized to the resonance intensity at 1 bar. The vast majority of residues displayed an intensity profile shown in black (group 1). White dots correspond to average normalized intensities at each value of pressure obtained for group 1. Near linear (group 2) and “mountain”‐like (group 3) pressure response of cross‐peak intensities obtained for two additional subsets of residues are shown in green (V37, T38, Q45, S50, Q51, H52, H57, G76, K77, K89, I103, S112) and pink (F2, E16, S25, K35, T73, F79, G88, F94, N96, Y97, H98, Q99, T113), respectively. Residues in group 1, 2, and 3 are mapped on the ribbon diagram of hI‐BABP (PDB: 1O1U) in the corresponding color. (b) ^1^H‐^15^N HSQC cross‐peak intensities relative to those at atmospheric pressure as a function of the amino acid sequence at 2000 and 2750 bar are shown in pink and dark magenta, respectively. Secondary structural elements are indicated at the top. Residues with a “mountain”‐like intensity profile are marked with an asterisk. Residues exhibiting over 40% and 50% loss in their backbone amide resonance intensities at 2000 bar are mapped on the ribbon diagram of hI‐BABP (PDB: 1O1U) in light and dark pink, respectively. Destabilized residue interactions are highlighted.

In addition to chemical shift, resonance intensity is the other major NMR parameter suitable for monitoring the unfolding process. An overlay of pressure‐induced intensity changes of backbone amide cross‐peaks in 2D ^1^H‐^15^N HSQC spectra are shown in Figure 5a. While most residues display similar profiles, the pressure response of hI‐BABP exhibits a significant degree of heterogeneity, with three characteristic intensity profiles. The most characteristic behavior, observed at approximately 70% of the analyzable amino acid positions, is a modest intensity decrease up to ~1500 bar, followed by a more pronounced decay up to 2750–3000 bar. A second group of residues (Figure 5a, green) exhibits an almost linear intensity decrease in the investigated pressure range. Finally, a third group of residues (Figure 5a, pink) displays a “mountain‐like” profile showing an initial gain in intensity with increasing pressure followed by a decrease. This anomalous behavior is observed primarily in the EFGH protein region, coinciding with segments undergoing pronounced ms‐timescale conformational fluctuations in *apo* hI‐BABP (Horváth et al., 2014 and cf below). Similar “mountain”‐like pressure response of resonance intensities, while unusual, has previously been reported for specific residues in ubiquitin (Kitahara & Akasaka, 2003) and in the outer surface protein A (OspA), a 31‐kDa immunogenic lipoprotein with an extended β‐sheet structure (Kitahara et al., 2012). As an explanation for the anomalous intensity changes, we hypothesize that, up to a certain pressure, line‐broadening arising from slow conformational exchange results in cross‐peak intensities that are lower than those of residues in less mobile segments on the ms‐timescale. As increasing pressure slows down the exchange (cf below), thereby reducing its contribution to transverse relaxation (i.e., line broadening), residues undergoing conformational exchange gain intensity up to a certain pressure. Residues with a “mountain”‐like intensity profile in hI‐BABP reach a plateau in the range of 1250–2000 bar followed by a decrease in resonance intensity similar to the rest of the protein reflecting increasing disorder in the polypeptide chain. Intensities of individual cross‐peaks at 2000 and 2750 bar, normalized to those at atmospheric pressure, are shown in Figure 5b as a function of the amino acid sequence. By 2750 bar, the population of the folded state decreases dramatically. To capture the onset of destabilization, residues exhibiting intensity decreases of 40% and 50% at 2000 bar are mapped onto the ribbon diagram of *apo* hI‐BABP (Figure 5b, right). In addition to perturbations of strand‐strand interactions, primarily between βA‐βB and βI‐βJ, the most affected region appears to be a hydrophobic cluster at the bottom of the β‐barrel, involving F47, F63, I71, V83, M85, and V92.

### 2.4 “Local” thermodynamic parameters of unfolding as assessed by resonance specific analysis of pressure‐induced cross‐peak intensity decays

**FIGURE 6:**
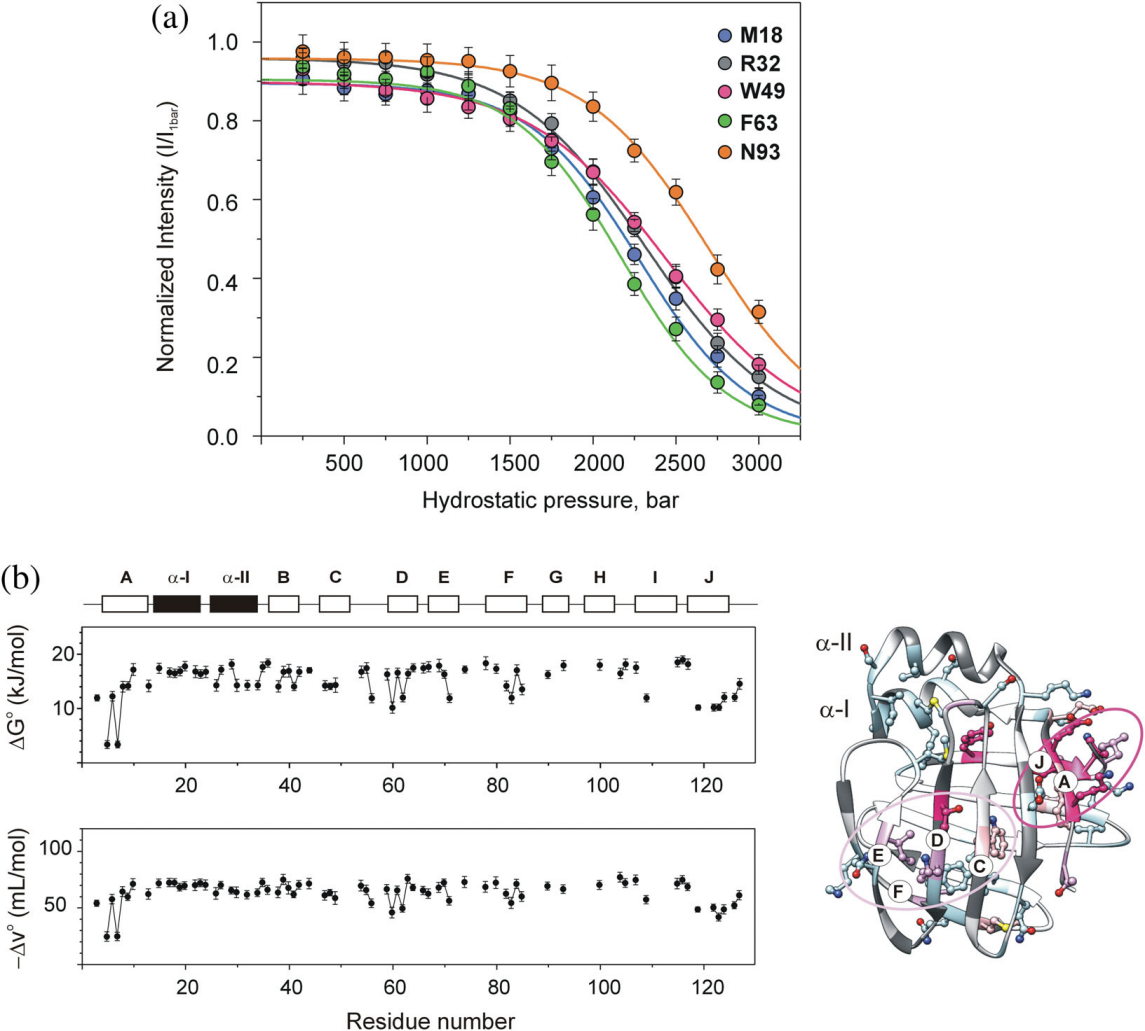
Thermodynamic analysis of the pressure response in hI‐BABP. (a) Normalized ^1^H‐^15^N HSQC cross‐peak intensity profiles measured at equilibrium for a representative set of residues in group 1 (black in Figure 5a), fitted with Equation (1). (b) Residue‐specific apparent local free energy changes (ΔG^o^) and apparent local molar volume changes (ΔV^o^) of unfolding, deduced by fitting the normalized intensity profiles. Secondary structural elements are indicated at the top. Residues exhibiting ΔV^o^ deviating from the average by more than one standard deviation are mapped on the ribbon diagram of the protein (PDB: 1O1U). Residue‐specific apparent local free energy and molar volume changes of pressure‐induced unfolding are listed in Table S1.

To characterize the pressure‐induced transition thermodynamically, cross‐peak intensities were analyzed using Equation (1). The analysis uses the important assumption that peak intensity for each residue can be interpreted as the preferential formation (or loss) of structure. Accordingly, cross‐peak intensities arising from the folded conformer reflect the population of the native state at each pressure. Representative intensity profiles fitted with Equation (1), assuming a two‐state transition, are shown in Figure 6a. Residues with less than eight data points or exhibiting anomalous behavior were excluded from the analysis. Local values of the apparent free energy change (ΔG^o^) and apparent molar volume change (ΔV^o^) of unfolding for the analyzable 70 residues averaged around 15.5 ± 2.4 kJ/mol and −65 ± 8 mL/mol, respectively, and are plotted in Figure 6b as a function of the amino acid sequence. The average values are comparable to those reported for titin I27 (Herrada et al., 2018), a protein of similar size and a variant of staphylococcal nuclease at ambient temperatures by high‐pressure NMR (Vidugiris et al., 1996). Residue‐specific “local” values of ΔG^o^ and ΔV^o^ deduced from individual fits are listed in Table S1. Heterogeneity in ΔV^o^ and ΔG^o^ in polypeptide chains indicates deviations from two‐state unfolding and highlights regions most susceptible to destabilization. Volume changes below the average by more than one |rmsd| were found in three specific regions of hI‐BABP such as (i) βA (K5, E7), (ii) the C/D‐turn (G56) and the D‐E region (T60, K62, I71), and (iii) βJ (Y119, S123‐K124, L126). Smaller but significant deviations were additionally observed for T3, W6, E9 (βA), W49 (βC), M85 (βF), and V109 (βI). Low in absolute value local volume changes were accompanied by below average local free energy changes, indicating that structure in these regions is disrupted more readily by pressure than in the rest of the protein.

### 2.5 Destabilization of residue‐residue contacts upon pressure increase assessed by fractional contact map analysis

**FIGURE 7:**
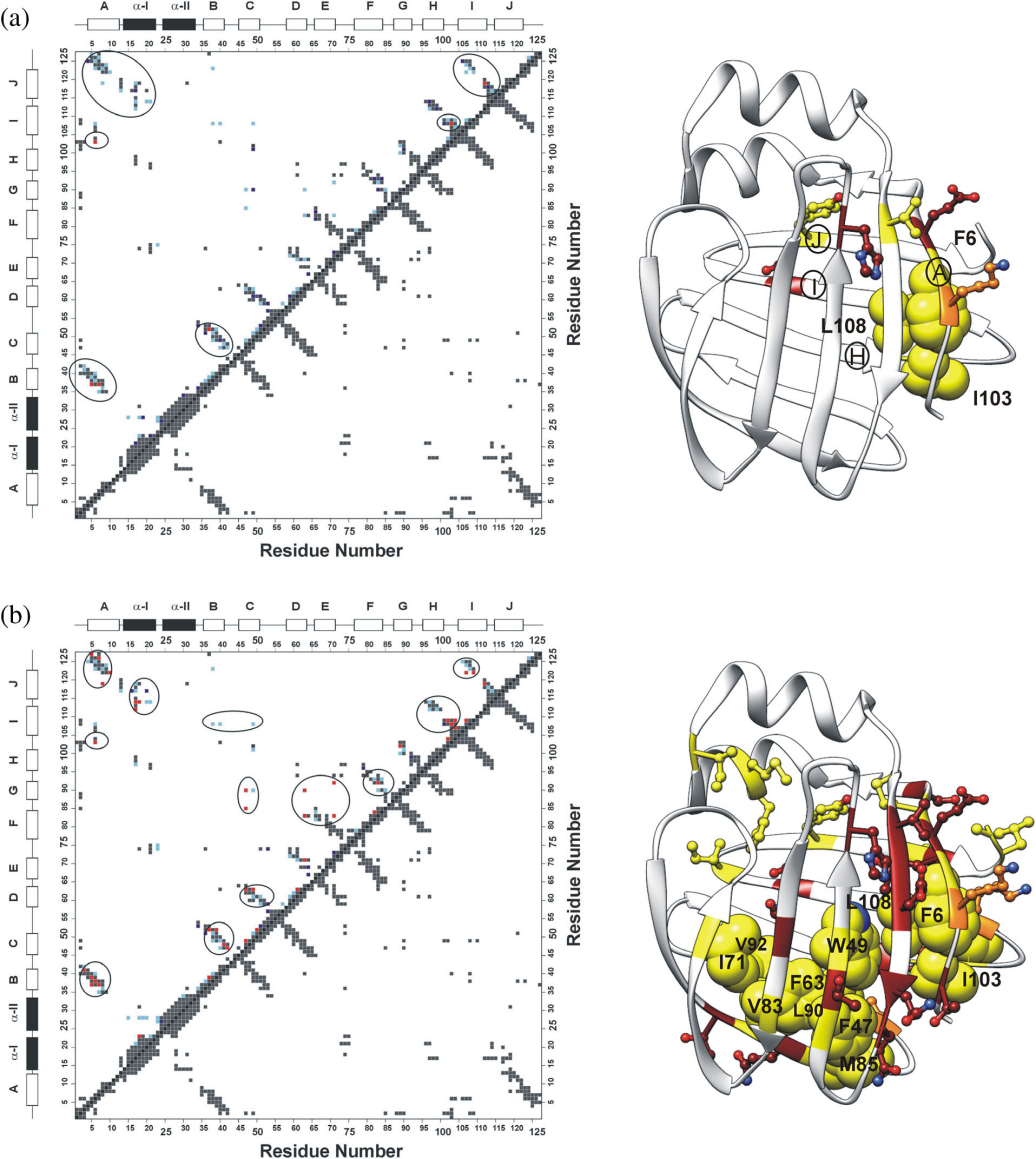
Pressure induced changes in native residue‐residue contacts in hI‐BABP deduced from high pressure NMR at (a) 2000 and (b) 2250 bar. Contact map was built based on the lowest‐energy solution NMR structure of hI‐BABP (PDB: 1O1U). Non‐sequential residue *i* and residue *j* were considered to be in a native contact if any side‐chain atom of residue *i* is within 4.0 Å of any side‐chain atom of residue *j*. All native contacts are shown below the diagonal. Above the diagonal, only those contacts are displayed for which a contact probability could be calculated from the corresponding residue‐specific cross‐peak intensities. Residues with resonance overlap and those displaying a “mountain”‐like intensity profile as a function of pressure have been omitted from the analysis. Color coding corresponds to contact probabilities (*v*_*ij*_) as follows: navy 0.6 < *v*(*i,j*) ≤ 0.7, cyan 0.5 < *v*(*i,j*) ≤0.6, red *v*_*ij*_ ≤ 0.5. Residues involved in pairwise contacts severely affected by pressure with *v*(*i,j*) ≤0.5 are mapped on the ribbon diagram of hI‐BABP. Residues are color coded according to their side chain as follows: Ala, Ile, Leu, Met, Phe, Trp, Tyr, Val in yellow, Asn, Asp, Gln, Glu, His, Thr in red, Arg, Lys in orange.

Pressure‐induced destabilization in hI‐BABP was further analyzed using fractional contact maps. A list of native contacts was first determined based on the solution NMR structure of *apo* hI‐BABP (PDB: 1O1U) (Kurz et al., 2003). For the analysis, non‐sequential residues *i* and *j* were considered to be interacting if any side‐chain atom of residue *i* is within 4.0 Å of any side‐chain atom of residue *j*. The pressure dependence of interresidue contacts was analyzed assuming that the probability of finding residue *i* in a folded state at a specific pressure (p) can be estimated by the corresponding normalized resonance intensity (I_i_^p^). Using this approach, the probability of forming a specific native contact between residues *i* and *j* can simply be calculated at each pressure as the geometric mean of the fractional intensities of the amide resonances v(i,j)^p^ = √I_i_^p^ * I_j_^p^ (Fossat et al., 2016). Contact maps for hI‐BABP reflecting the presence/absence of native inter‐residue contacts at 2000 and 2250 bar are shown in Figure 7. The first region affected by pressure increase is the N‐terminal β‐strand (βA). Additionally, localized strand‐strand interactions become destabilized in both the N‐ and the C‐terminal β‐sheets. Notably, this includes a small cluster of hydrophobic residues (F6(βA), I103(βH), L108(βI)), whose side chains form stabilizing interactions in the native state. Upon further increasing the pressure, perturbations are propagated by strand‐strand interactions to the bottom of the β‐barrel and to the interface between the I/J‐turn and the helical cap. At the bottom of the β‐barrel, significant loss of native contacts (v(i,j)^*p*^ < 50%) occurs between βC and βG (F47‐L90, W49‐L90), βC and βF (F47‐M85), βD and βF (F63‐V83), and between the D‐E region and βG (F63‐L90, I71‐V92). Remarkably, the secondary structure of the α‐helices is largely unaffected even at 2250 bar. However, native contacts between α‐I and the I‐J region begin to become destabilized.

### 2.6 Slow conformational fluctuations at atmospheric and high pressure by CPMG relaxation dispersion analysis

**FIGURE 8:**
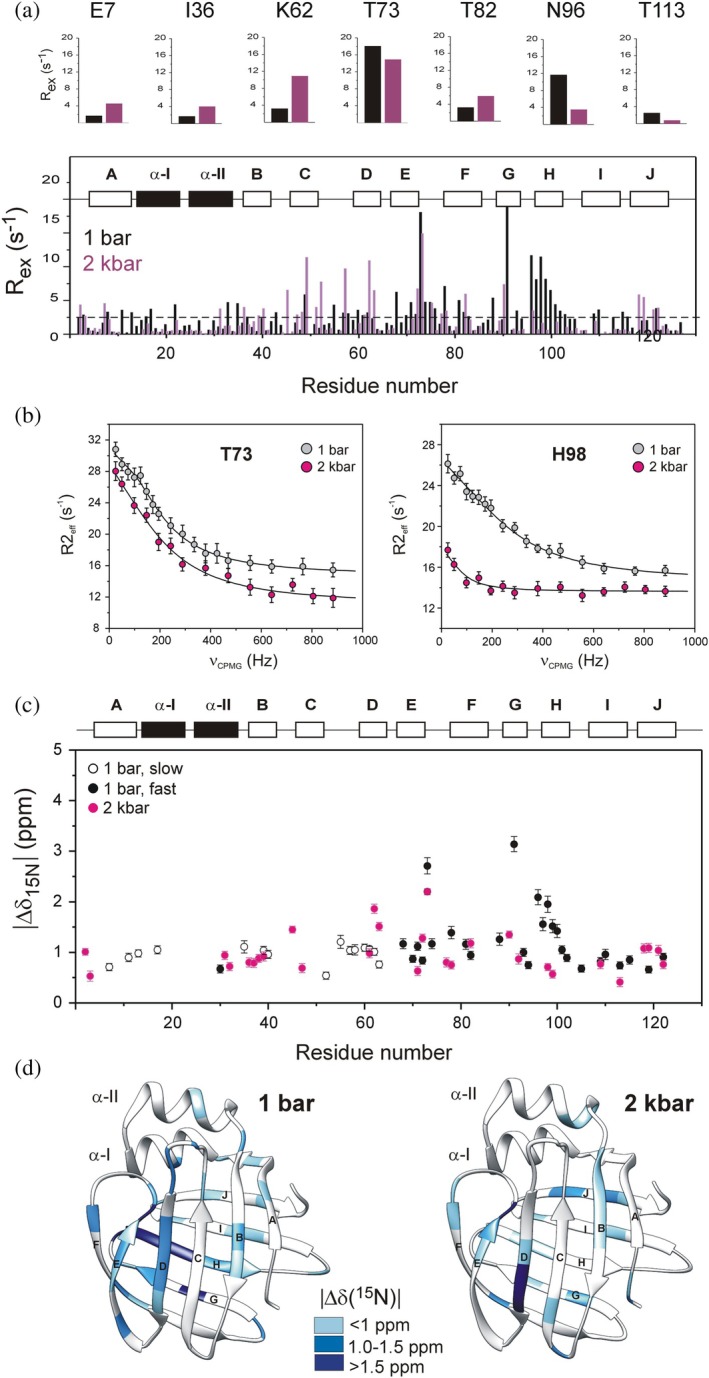
Slow conformational fluctuations in hI‐BABP at atmospheric and high pressure. (a) Contribution to transverse relaxation from conformational exchange in ^2^H/^15^N‐labeled *apo* human I‐BABP at 18°C and 1 bar (gray) and 2000 bar (pink) as a function of the amino acid sequence. Values of R_ex_ were estimated from the difference in R_2eff_ at the lowest and highest ν_CPMG_ values. Secondary structural elements are indicated at the top. For representative residues, values of ^15^N R_ex_ at the two investigated pressures are highlighted at the top. (b) Transverse relaxation dispersions of the backbone ^15^N nuclei (at a static magnetic field strength of 14.1 T) of two representative residues in *apo* hI‐BABP as a function of CPMG B_1_ field strength at 1 bar (gray) and 2000 bar (pink) (18°C, pH = 6.3). Solid black lines correspond to global two‐state exchange models with parameters listed in Table 1. (c) Backbone ^15^N chemical shift differences between the ground and the higher energy state (|Δδ_i_|) as derived from CPMG relaxation dispersion measurements on *apo* hI‐BABP at 1 bar (white: “slow” group, black: “fast” group) and 2000 bar (pink) as a function of the amino acid sequence. (d) Values of |Δδ_i_| are mapped on the ribbon representation of the lowest energy solution structure of hI‐BABP (PDB entry 1O1U) for both pressures with color coding depicted in the figure. Residues exhibiting a flat dispersion profile or with no available data are in white. Chemical shift differences between the ground and the higher energy state at the two investigated pressures are listed in Tables S2, S3.

To gain insight into the structural characteristics of hidden conformers with functional implications, the pressure dependence of the previously identified global conformational exchange in *apo* hI‐BABP (Horváth et al., 2012, 2014, 2016, 2019) was examined. CPMG relaxation dispersion NMR measurements suitable for unveiling the kinetic, thermodynamic, and structural aspects of conformational equilibria between the ground and sparsely populated higher energy states (Loria et al., 1999; Mulder et al., 2001; Tollinger et al., 2001) were performed at atmospheric pressure and at 2000 bar, where major unfolding begins. Contributions from ms‐timescale conformational fluctuations to transverse relaxation are shown in Figure 8a. Representative transverse relaxation dispersions as a function of the CPMG field strength are plotted in Figure 8b. Dispersion profiles were fit assuming two‐state exchange as described in Materials and Methods. At atmospheric pressure, measurements under the current experimental conditions have shown a good agreement with previously reported data (Horváth et al., 2014). Specifically, two groups of residues with slightly different exchange rates were observed involving segments of the C‐terminal β‐sheet, mainly the EFGH protein region (“fast group”), and part of the helical cap and segments of the N‐terminal β‐sheet (“slow group”). Conformational exchange at 2000 bar was observed in similar regions, with the exception of α‐I, which remained silent on the CPMG‐sensitive timescale (0.3–10 ms). Importantly, slow fluctuations become more prevalent in the CD‐region and in βJ, whereas they are markedly reduced in the GH‐region at 2000 bar. As expected, conformational fluctuations slowed down at high pressure. Backbone amides undergoing conformational exchange at 2000 bar showed similar exchange rates, justifying a global fit (Table 1). Moreover, the two groups observed at 1 bar (k_ex_ = 1490 ± 135 s^−1^ and k_ex_ = 862 ± 102 s^−1^) merged into k_ex_ = 427 ± 71 s^−1^ at high pressure. The population of the higher energy state at 2000 bar (p_b_ = 3.8% ± 0.2%) was similar to the fast (p_b_ = 4.4% ± 0.2%) and slow (p_b_ = 2.2% ± 0.2%) groups at 1 bar. Chemical shift differences inferred from CPMG measurements between the exchanging states for individual ^15^N spins are shown in Figure 8b,c. While in most segments of the protein values of Δδ_15N_ were similar at high pressure to those at 1 bar, the G–H region exhibited a marked decrease in Δδ_15N_, indicating a smaller structural difference between the ground and excited states at high pressure. In the C‐terminal half of βD (near the D/E‐turn), residues K62 and F63 showed increased Δδ_15N_ upon pressure increase. Detailed dynamic and structural parameters from the global fit analysis, including residue‐specific chemical shift differences between the exchanging states, are provided in Tables S2, S3.

**TABLE 1:** Kinetic and thermodynamic parameters of conformational exchange in *apo* human I‐BABP at 1 bar and 2000 bar (18°C, pH = 6.3) deduced from global fit analysis of ^15^N backbone relaxation dispersion NMR measurements assuming a two‐state (A ↔ B) exchange process between the ground and a higher energy state.

	1 bar		2000 bar
	Group I	Group II	
k_ex_ (s^−1^)	862 ± 102	1490 ± 135	427 ± 71
p_B_ (%)	2.2 ± 0.2	4.4 ± 0.2	3.8 ± 0.2
k_AB_ (s^−1^)	19 ± 3	66 ± 7	16 ± 3
k_BA_ (s^−1^)	843 ± 126	1424 ± 144	411 ± 72
ΔG_AB_ (kcal/mol)	2.2 ± 0.1	1.8 ± 0.1	1.9 ± 0.1

*Note*: At 2000 bar, the two groups of residues observed at atmospheric pressure merge with a single exchange rate.

## 3 DISCUSSION

Hidden protein states with partially unfolded regions can have functional relevance and may also contribute to protein aggregation and amyloid formation. By shifting the thermodynamic equilibrium toward lower‐volume states, high pressure tends to promote the dissociation of protein aggregates into monomeric species. This can be exploited in the study of amyloidogenic proteins and other, predominantly disordered polypeptide chains, in which transiently formed structured regions may serve as nucleation points for aggregation (Barnes et al., 2019; Munte et al., 2013; Rosenman et al., 2018; Vemulapalli et al., 2021). Moreover, high pressure NMR can be used to identify sparsely populated conformational states coexisting with the major state under normal pressure in highly dynamic globular systems through the observation of their pressure response (Girard et al., 2022; Kalbitzer et al., 2013).

Bile acid‐binding proteins, a subfamily of iLBPs, are thought to exploit order–disorder transitions and low‐populated higher‐energy conformers in ligand uptake/release and ligand‐mediated stimulation of the transcriptional activity of nuclear receptors. This study, through a residue‐specific NMR spectroscopic investigation of the pressure response, provides new insights into the determinants of stability and the folding/unfolding mechanism of human I‐BABP. Furthermore, NMR relaxation analysis of slow internal motions at atmospheric and high pressure improves our understanding of functionally relevant conformational fluctuations, with implications for the role of partially unfolded states in ligand recognition.

In agreement with previous kinetic and structural studies of related iLBPs using chemical denaturation, high pressure NMR suggests a complex unfolding mechanism for hI‐BABP. In seeking the structural origin of deviations from two‐state unfolding, residues in four main protein regions, such as the N‐terminal β‐strand, the helical cap, the bottom of the β‐barrel, and segments of the HIJ region, exhibit pressure responses that differ significantly from the rest of the protein. Among these, βA and proximate segments of βH/βI/βJ are most susceptible to pressure increase. Specifically, a small cluster of residues involved in conserved pairwise interactions (F6‐I103, F6‐L108, F6‐R125), which clamp together the N‐ and C‐terminal peripheral segments of the protein, appears to be the first to become destabilized at the onset of the main transition. Among them, F6 and L108 additionally form hydrophobic contacts with V40 (βB) and W49 (βC) in the native state, propagating pressure‐induced structural changes toward the B‐C region. Structural rearrangement in this region is consistent with the appearance of an intermediate resonance between the folded and unfolded peaks of the W49 side‐chain proton (Figure 2). Vulnerability to pressure increase of long range interactions involving βA and the IJ‐region is also reflected in low apparent local volume changes accompanying the unfolding process (Figure 6b). The role of the peripheral N‐terminal region in the stability of hI‐BABP is further indicated by the pressure response of F2, which, engaged in several conserved hydrophobic contacts in the native state (Figure S3), experiences above average changes in the chemical environment near the main transition (~2500 bar). In addition, NMR relaxation measurements indicate that F2 and T3 undergo ms‐timescale conformational fluctuations at the onset of the main transition, consistent with partial unfolding and formation of an intermediate. The susceptibility of βA to high hydrostatic pressure is in agreement with urea denaturation studies of I‐FABP, where the vicinity of W6 unfolds at denaturant concentrations at which other regions still retain a residual structure (Dalessio et al., 2005).

Following destabilization of the peripheral βA‐βH/βI/βJ contacts, specific segments at the bottom of the β‐barrel, involving residues from several β‐hairpins, exhibit marked intensity decreases at pressures where major unfolding begins. This includes a cluster of residues with bulky aromatic and aliphatic side chains in the hydrophobic core, which lose pairwise contacts around 2250 bar (Figure 7). Most of these interactions are conserved in iLBPs (F47‐F63, F63‐L90, V83‐L90) and form part of a larger network involving βA (F2, F6), βB (V40), βC (W49), βH (I103), and βI (L108) (Figure S3). In addition to hydrophobic interactions, W49, L90, and L108 form hydrogen bonds with the backbone atoms of N61, S101, and S123 (Horváth et al., 2016; Kurz et al., 2003), contributing to the stability of βD, βH, and βJ, respectively. Residue‐specific analysis shows that loss of native contacts at the bottom of the β‐barrel initiates an overall disintegration of the β‐fold, evidenced by a marked loss of resonance intensity corresponding to the folded state and an increase of the disordered population (Figures 3, 5). Consistent with previous ^19^F NMR studies of urea denaturation (Basehore & Ropson, 2011), F63 emerges as a key residue of the hydrophobic core in hI‐BABP and likely in other iLBPs as well (Ropson & Frieden, 1992). While experiencing large changes in the chemical environment between 2250 and 3000 bar (Figure 4b), F63 is part of a continuous stretch of residues (F63‐N70) exhibiting relatively high local stability (Table S1). Considering the fully reversible nature of pressure‐induced unfolding, this might be an indication of the involvement of F63 and the proximate D/E‐turn in an early folding step, that is, formation of a nucleation site. This is consistent with the formation of a template of tertiary interactions preceding β‐sheet formation, a model previously proposed for IFABP (Dalessio et al., 2005; Kim et al., 1997; Li & Frieden, 2005a, 2005b; Ropson & Frieden, 1992), suggesting that this may represent a common mechanism in iLBPs, as indicated by two distinct methodologies, that is, chemical denaturation (I‐FABP) and high hydrostatic pressure (hI‐BABP).

Between 2250 and 3000 bar, the hydrophobic cluster at the bottom of the β‐barrel and segments of the peripheral EF‐region display a concerted decrease in resonance intensity, indicating highly cooperative destabilization. NMR relaxation measurements highlight the flexible nature of this protein region in BABPs on both fast (ps‐ns) and slow (ms) timescales (Horváth et al., 2012, 2014; Ragona et al., 2006). The plasticity of the region likely contributes to adaptability in ligand transfer (Pedò et al., 2009) and may have implications for the exposure of specific hydrophobic residues involved in signaling (Ayers et al., 2007; Sessler & Noy, 2005). Additionally, flexibility at the bottom of the β‐barrel may be related to hosting a nucleation site during folding, requiring sufficient fluidity to guide subsequent folding steps (Li & Frieden, 2007a, 2007b).

The protein region most resistant to high hydrostatic pressure in hI‐BABP is the helical cap. It is one of the most intriguing structural elements in iLBPs, whose role in function and stability remains a subject of debate. In I‐FABP, removal of the helical region has little effect on the rate of β‐barrel formation and its stability (Cistola et al., 1996; Kim et al., 1996; Steele et al., 1998), whereas a helix‐less mutation of *apo* rabbit I‐BABP unfolds under physiological conditions (Kouvatsos et al., 2006). The role of the helical cap in ligand binding also varies across iLBP subfamilies. In FABPs, stabilizing interactions between the helical segment and the C/D‐loop are thought to be the key determinants of an order–disorder transition regulating ligand entry (Hodsdon & Cistola, 1997a). In BABPs, NMR relaxation measurements indicate an alternate portal in the EFGH region that mediates ligand entry via a conformational selection mechanism (Horváth et al., 2014, 2016, 2019; Ragona et al., 2006). Intriguingly, in the human analogue, NMR relaxation measurements at atmospheric pressure show evidence of a second group of residues undergoing conformational exchange with a somewhat slower exchange rate overlapping with the helical cap and the C/D‐loop, that is, the dynamic portal in FABPs (Hodsdon & Cistola, 1997a). Accordingly, an equilibrium between an EFGH‐closed/α‐open and an EFGH‐open/α‐closed conformation has been proposed to preexist in *apo* hI‐BABP, shifting upon ligand binding (Horváth et al., 2016, 2019).

An important difference between iLBP subfamilies is the flexibility of the helical cap. In FABPs and some of the reported CRBPs, the helical region is considerably disordered in the *apo* state (Franzoni et al., 2002; Lu et al., 2003; Menozzi et al., 2018), whereas in BABPs it is well defined in both the free and the bound forms (Ragona et al., 2014). Pressure denaturation supports the compact, rigid nature of the hI‐BABP helical cap, remaining well‐defined even at pressures where the rest of the protein is destabilized. Unlike in IFABP (Hodsdon & Cistola, 1997b) and CRBP‐II (Lu et al., 2003), no significant differences appear to exist between the two helices in terms of local stability. However, α‐I forms extensive hydrophobic contacts with the I‐J β‐strands (F17/L20‐I114/V117, Y14‐Y119), which are maintained up to ~2250 bar but begin to become destabilized during the main transition, suggesting a critical role in hI‐BABP stability. A possible role of α‐I in BABP folding/unfolding has also been indicated by ^19^F NMR showing a large chemical shift change for F17 at urea concentrations where most of the protein is already unfolded (Basehore & Ropson, 2011).

Unfortunately, some segments of βE and the G‐H region display anomalous “mountain”‐like intensity profiles due to conformational exchange, hindering a full analysis. Notably, protein regions with above average pressure‐induced fluctuations in the folded manifold overlap with segments exhibiting a prevalent conformational exchange, suggesting a link between pressure‐induced structural alterations in the pretransition range and a hidden conformer revealed by relaxation dispersion measurements. We note that the CPMG‐derived higher energy state in hI‐BABP has previously been associated with temperature‐induced unfolding (Horváth et al., 2017). Specifically, protein regions exhibiting the most pronounced conformational exchange have been found to overlap with segments that show a nonlinear ^1^H‐^15^N chemical shift pattern as a function of temperature, characteristic of a multistate unfolding process. Furthermore, residues which display large‐amplitude relaxation dispersions have been found to exhibit large ^15^N chemical shift differences between deconvoluted folded and partially unfolded states in NMR thermal melts (Horváth et al., 2017). The present study provides new indications of the partially unfolded nature of the higher energy state in the portal region of *apo* hI‐BABP. Specifically, as reflected by the chemical shift differences between the exchanging states at atmospheric and high pressure, the ground and higher‐energy states become less distinct in the EFGH‐region (the proposed portal region) at the onset of the main transition. This is consistent with our hypothesis (Horváth et al., 2016, 2017) that conformational exchange between a closed and a more open EFGH‐region mediating ligand entry is related to a partial unfolding of hI‐BABP. Acting as a hinge, the D/E turn‐region may play an important role in the opening‐closing motion of the EF‐hairpin. CPMG R_ex_ measurements showing increased contribution to transverse relaxation at the C‐terminal end of βD (K62‐F63) at 2000 bar further support the link between conformational exchange and pressure‐induced structural alterations.

**FIGURE 9:**
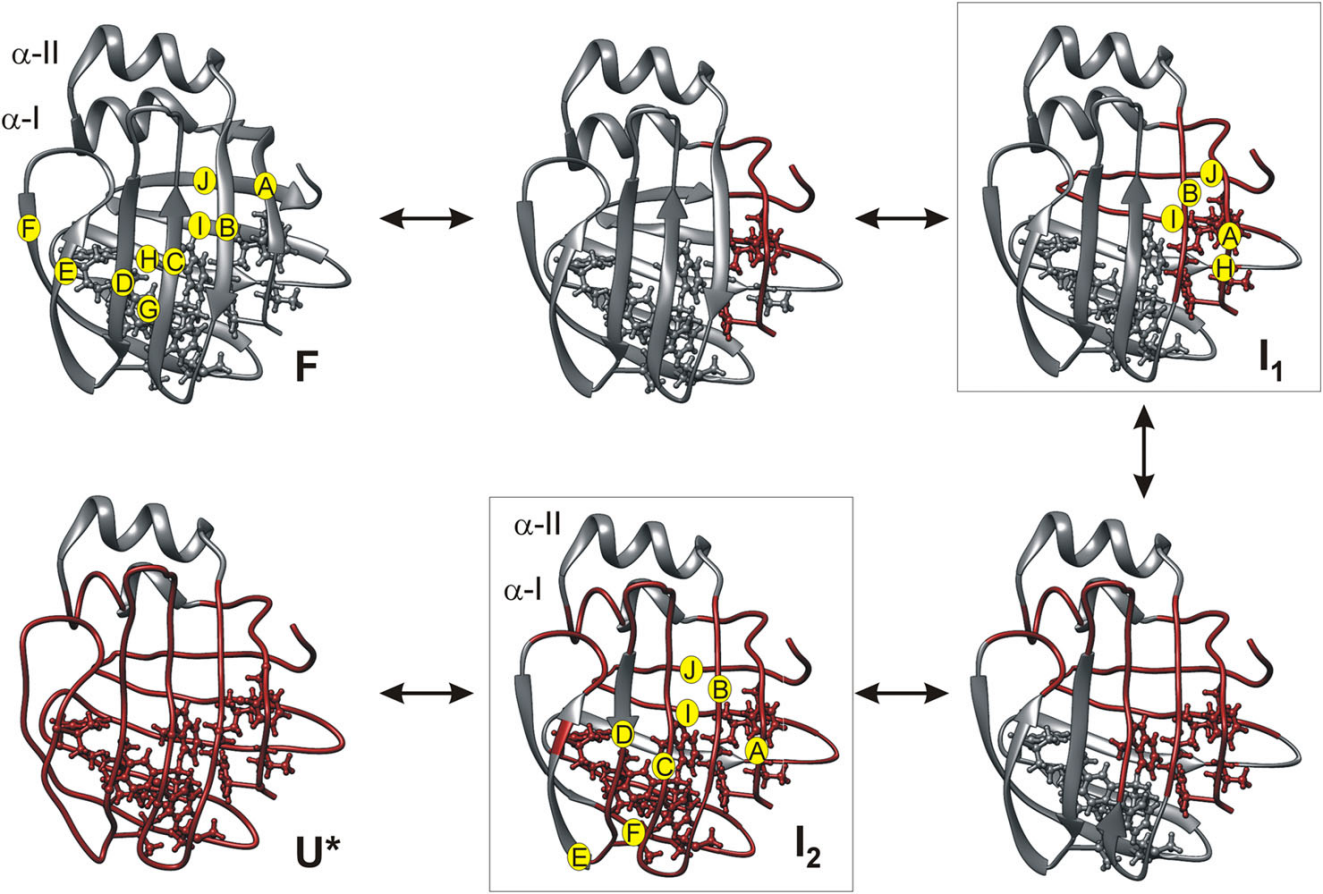
Proposed mechanism of pressure‐induced unfolding of hI‐BABP. Unfolding begins with destabilization of the peripheral βA strand and the neighboring segments of βH, βI, and βJ, followed by the propagation of disorder toward the central segments of the β‐strands in both the N‐ and C‐terminal β‐sheets. By strand‐strand interactions, the disorder reaches the hydrophobic core at the bottom of the β‐barrel resulting in an overall loss of structural integrity. Residual structure in the helical cap is preserved but packing interactions between α‐I and βI/βJ begin to be compromised. Possible unfolding intermediate(s) are highlighted.

Based on the observed pressure response, we propose a pathway of destabilization, as illustrated in Figure 9. Unfolding of hI‐BABP begins with the destabilization of βA and loosening of its contacts with βB. In parallel, proximate segments of the HIJ β‐strands begin to become destabilized. Upon the loss of hydrophobic contacts in the region, disorder propagates toward the central segments of the affected β‐strands in both the N‐ and C‐terminal β‐sheets. As disorder reaches the hydrophobic core of the protein, the bottom of the β‐barrel becomes increasingly destabilized affecting segments in different β‐strands and extending toward the flexible E/F‐region. Contacts between α‐I and the I‐J strands are concomitantly compromised. The observed spectral changes and the heterogeneity in pressure response indicate a multistate unfolding process involving at least one, or possibly two, intermediates (Figure 9). Considering the reversibility of the unfolding process, the proposed pathway of destabilization is consistent with a folding mechanism in which hydrophobic collapse initiates folding, followed by β‐strand formation and stabilization of peripheral segments by long‐range side‐chain interactions. The high stability of the helical cap suggests that the helices form independently of the β‐barrel at the early stages of folding and hydrophobic contacts between α‐I and specific residues in the I‐J region facilitate the formation of the native barrel.

The pathway of destabilization and the proposed folding mechanism support the role of turns in folding initiation (Rose, 2025). Specifically, native‐like nucleation centers at specific turn regions preexisting in the unfolded state are expected to reduce the entropic cost of the formation of β‐hairpins, which then assemble into a larger cluster. The pressure response of the hydrophobic cluster at the bottom of the β‐barrel highlights the importance of B/C‐ and D/E‐turns in the stability of hI‐BABP, consistent with the presence of nascent β‐turns at the early steps of folding (Hodsdon & Frieden, 2001; Nikiforovich & Frieden, 2002). The concerted pressure response of hydrophobic clusters, comprised of residues distant in sequence, also shares common features with a mechanism involving the formation of larger loops during the initial stages of folding (Berezovsky et al., 2001; Bergasa‐Caceres et al., 2019). In addition to the bottom of the β‐barrel, such loops involving specific segments of the HIJ‐region may set the stage for stabilizing interactions with the N‐terminal β‐strand and α‐I.

In conclusion, residue‐specific analysis of the pressure response of hI‐BABP identifies several long‐range interaction clusters that are critical for protein stability. These clusters likely contribute both to early folding events involving the bottom of the β‐barrel and the helical cap, as well as to the stabilization of peripheral segments of the protein during later stages of folding. As indicated by chemical shift and relaxation dispersion analysis, pressure‐induced conformational fluctuations in the pretransition range are likely related to functionally relevant conformational exchange in the folded state. Site‐directed mutagenesis planned for the future should shed more light on the relationship between pressure‐induced structural alterations and slow internal dynamics in the native state. Meanwhile, relaxation dispersion measurements carried out at high pressure provide new indications for the partially unfolded nature of the higher‐energy hidden state with implications for ligand transfer and bile salt mediated signaling processes in hI‐BABP. The identified residues and protein segments that play a key role in governing the pressure response may facilitate the development of iLBP‐based contrast agents (D'Onofrio et al., 2012; Tomaselli et al., 2008) and drug delivery systems (Chuang et al., 2008; González & Fisher, 2015; Patil et al., 2014) with enhanced stability.

## 4 MATERIALS AND METHODS

### 4.1 Protein biosynthesis and purification

For uniformly ^15^N‐enriched protein, the *Escherichia coli* strain MG1655, transformed with the pMON5840‐hIBABP construct, was incubated overnight (37°C, 250 rpm) in 50 mL non‐isotope‐enriched minimal media (6 g/L Na_2_HPO_4_, 3 g/L KH_2_PO_4_, 0.5 g/L NaCl, 1 g/L NH_4_Cl, 1 mM MgSO_4_, 5 g/L D‐glucose, 25 mg/L thiamine.HCl, 0.1 mM CaCl_2_) and trace metals according to Li et al. (1987) containing 100 μg/mL ampicillin, then transferred into a 2‐L flask, containing 700 mL fresh isotope‐enriched minimal media with 0.7 g ^15^NH_4_Cl. Protein expression, under the control of the recA promoter, was induced by the addition of 7 mL 10 mg/mL nalidixic acid to the growing culture at OD_600_ ~ 2.0 and cells were allowed to grow 3–4 h more, harvested, and frozen at −80°C. For the uniformly (80% ^2^H, 99% ^15^N) enriched hI‐BABP, a one‐stage protocol was applied. Specifically, cells were grown in 800 mL of minimal media in 99% D_2_O supplemented with ^15^NH_4_Cl at 37°C for 20 h, induced at an OD_600_ of ∼2.3, and grown for an additional 4 h before being harvested. Protein purification started with thawing the cell pellet in ~10–20 mL of 20 mM Tris‐Cl, pH 8.0, 5 mM EDTA, containing a broad‐spectrum protease inhibitor mixture (Roche Molecular Biochemicals). The protein was released by processing the cell suspension through an Emulsiflex C5 homogenizator (Avestin) 3–4 subsequent times. The homogenized cell suspension was subjected to centrifugation at 4°C, 23,000 g for 30 min. The clarified supernatant was chromatographed on a 25 × 5 cm column of Q‐Sepharose Fast Flow. Dialysis was carried out against multiple changes of 10 mM Tris, pH = 8.2, 0.05% NaN_3_ at 4°C, until the A_260_/A_280_ absorbance ratio decreased below 0.7 followed by gel filtration chromatography using a 110 × 2.5 cm column of Sephadex G‐50. Delipidation was achieved by passing the protein solution over a column of lipophilic Sephadex type VI (Sigma product no. H‐6258) pre‐equilibrated with 20 mM potassium phosphate, 50 mM KCl, and 0.05% NaN_3_ (pH 6.3), at 37°C (Glatz & Veerkamp, 1983). Protein purity, as assessed by overloaded Coomassie‐stained SDS‐PAGE gels, was >98%. Protein concentration was determined by absorbance at 280 nm using an extinction coefficient of 12,930 M^−1^ cm^−1^ obtained by composition analysis according to Pace et al. (Pace et al., 1995).

### 4.2 NMR data collection and analysis

One‐dimensional (1D) ^1^H (Scott et al., 1997) and two‐dimensional (2D) ^1^H‐^15^N heteronuclear single quantum coherence (HSQC) (Kay et al., 1992) experiments were carried out on a 600 *Varian NMR SYSTEM™* spectrometer equipped with a 5‐mm indirect detection triple (^1^H^13^C^15^N) z‐axis gradient probe at 18°C at the following pressures: 1, 250, 500, 750, 1000, 1250, 1500, 1750, 2000, 2250, 2500, 2750, and 3000 bar. Hydrostatic pressure was applied to the sample directly within the magnet using the Xtreme Syringe Pump (Daedelus Innovations). Initially, one‐dimensional (1D) NMR experiments were recorded at each pressure, monitoring the chemical shift and intensity changes of methyl peaks and the W49 side‐chain NH, diagnostic of the folding/unfolding process (Horváth et al., 2017). Based on the 1D measurements, an incubation time of 90 min was established to allow the system to reach full equilibrium and used subsequently before collecting the 2D ^1^H‐^15^N HSQC spectrum at each pressure. 1D ^1^H and 2D ^1^H‐^15^N HSQC spectra recorded at 1 bar before and after the pressurization series gave the same results, demonstrating the full reversibility of the folding/unfolding transition (Figures S1, S2).

^1^H‐^15^N HSQC spectra were acquired with spectral widths of 9615 and 1900 Hz in the ^1^H and ^15^N dimensions, respectively. The number of complex points in the ^1^H dimension was 2048, subsequently zero‐filled to a total of 4096 points. The number of increments in the indirectly detected dimension was 256, subsequently linearly predicted to 512. Gaussian and exponential weighting functions were applied in the F2 (^1^H) dimension, whereas the F1 (^15^N) dimension was Gaussian weighted only. Measurements were carried out in a buffer containing 20 mM potassium phosphate, 50 mM KCl, and 0.05% NaN_3_ at pH = 6.3 with a protein concentration of 400 μM. The ^1^H chemical shifts were referenced externally to 2,2‐dimethylsilapentane‐5‐sulfonic acid (DSS), whereas the ^15^N chemical shifts were referenced indirectly to DSS (Markley et al., 1998).

The intensities of amide cross‐peaks in 2D ^1^H‐^15^N HSQC spectra were measured for the folded species at each pressure and fitted with a two‐state model (Dubois et al., 2020):

(1) $$I=\frac{I_{u}+I_{f}\exp\left(-\frac{{∆G}_{f}^{0}+{p∆V}_{f}^{0}}{\mathrm{RT}}\right)}{1+\exp\left(-\frac{{∆G}_{f}^{0}+{p∆V}_{f}^{0}}{\mathrm{RT}}\right)}$$

where *I* is the cross‐peak intensity measured at a given pressure (p), *I*_f_ and *I*_u_ correspond to the cross‐peak intensities in the folded and in the unfolded states, respectively, and Δ𝐺_f_^0^ and ΔV_f_^0^ are the apparent free energy and apparent molar volume changes of folding at atmospheric pressure for a given residue. Creation of the contact map for the fractional contact map analysis at different pressures relied on the solution NMR structure of *apo* hI‐BABP (PDB: 1O1U) (Kurz et al., 2003).

Relaxation dispersion data at different values of hydrostatic pressure were obtained using a relaxation compensated Carr‐Purcell‐Meiboom‐Gill (CPMG) dispersion experiment performed in a constant time manner (Loria et al., 1999; Tollinger et al., 2001). The constant time delay (*T*_CP_) was set to 40 ms. Spectra were collected as a series of 19 two‐dimensional data sets with CPMG field strengths (*υ*_CPMG_) of 25, 50, 74, 99, 123, 147, 172, 195, 219, 242, 289, 335, 380, 425, 469, 556, 641, 764, and 883 Hz. A reference spectrum was obtained by omitting the CPMG period in the pulse sequence (Mulder et al., 2001). Spectra (3 s interscan delay, 32 transients) were acquired in duplicate. Contributions to transverse relaxation rates of conformational exchange in CPMG relaxation dispersion measurements were analyzed assuming a two‐state exchange process using the all‐timescales multiple‐quantum Carver‐Richards‐Jones formulation (Korzhnev et al., 2004) implemented in GUARDD (Kleckner & Foster, 2012). Molecular figures were generated using Chimera 1.10.2 (Pettersen et al., 2004).

## AUTHOR CONTRIBUTIONS

**Bence Balterer:** Investigation; methodology; visualization. **Orsolya Toke:** Writing – review and editing; writing – original draft; validation; supervision; methodology; investigation; data curation; conceptualization. **Tamara Teski:** Writing – original draft; visualization; investigation; formal analysis; data curation. **Gábor Turczel:** Methodology; data curation. **Gergő Horváth:** Data curation; methodology.

## CONFLICT OF INTEREST STATEMENT

The authors declare no competing interests.

## Supporting information


**Figure S1.** Reversibility of hI‐BABP pressure denaturation. (a) ^1^H‐^15^N HSQC NMR spectra of ^15^N‐labeled hI‐BABP (0.4 mM, 18°C, pH = 6.3) at atmospheric pressure recorded before (black) and after (cyan) the measurement series in the 1–3000 bar pressure range. The spectrum in cyan is shifted upward for better viewing. The spectrum in gray was collected at 3000 bar. (b) One‐dimensional ^1^H‐NMR spectra of the same sample showing the full ^1^H spectral range at atmospheric pressure before (black) and after (cyan) the completion of the pressurization series.
**Figure S2.** Reversibility of hI‐BABP pressure denaturation. (a) ^1^H and (b) ^15^N chemical shift differences as a function of the amino acid sequence as determind from ^1^H‐^15^N HSQC NMR spectra of ^15^N‐labeled hI‐BABP (Figure S1) recorded at atmospheric pressure before and after the measurement series in the 1–3000 bar pressure range. (c) Ratio of peak intensities as a function of amino acid sequence between the two measurements at 1 bar.
**Figure S3.** (a) Sequence alignment of human I‐BABP and rat I‐FABP discussed in the main text. Residue numbering is according to hI‐BABP. Secondary structural elements indicated at the top are according to *apo* hI‐BABP. The break occurring in βF is indicated in gray. (b) Conserved pairwise interactions in human I‐BABP (PDB: 1O1U; Kurz et al., 2003, left) and rat I‐FABP (PDB: 1AEL; Hodsdon & Cistola, 1997a, right). Short‐ and long‐range interactions are depicted in dashed and solid lines, respectively. Residues involved in short‐range, long‐range, or both short‐ and long‐range interactions are depicted in yellow, blue, and magenta, respectively. Residues involved in multiple long‐range interactions are marked with a green dot. Note the missing residues in panel (a) affecting the numbering in panel (b).
**Table S1.** “Local” thermodynamic parameters of pressure‐induced unfolding obtained for *apo* hI‐BABP in 20 mM K‐phosphate, 50 mM KCl, 0.05% NaN_3_, pH = 6.3 at 291 K.
**Table S2.** Exchange parameters derived from global fit analysis of ^15^N relaxation dispersion curves assuming a two‐state exchange obtained for *apo* hI‐BABP in 20 mM K‐phosphate, 50 mM KCl, 0.05% NaN_3_, pH = 6.3 at 291 K, 1 bar.
**Table S3.** Exchange parameters derived from global fit analysis of ^15^N relaxation dispersion curves assuming a two‐state exchange obtained for *apo* hI‐BABP in 20 mM K‐phosphate, 50 mM KCl, 0.05% NaN_3_, pH = 6.3 at 291 K, 2000 bar.

## Data Availability

Data and materials are available from the corresponding author upon request.

## References

[pro70608-bib-0001] Akasaka K . Probing conformational fluctuation of proteins by pressure perturbation. Chem Rev. 2006;106:1814–1835. 16683756 10.1021/cr040440z

[pro70608-bib-0002] Ayers SD , Nedrow KL , Gillilan RE , Noy N . Continuous nucleocytoplasmic shuttling underlies transcriptional activation of PPARγ by FABP4. Biochemistry. 2007;46:6744–6752. 17516629 10.1021/bi700047a

[pro70608-bib-0003] Banaszak L , Winter N , Xu Z , Bernlohr DA , Cowan S , Jones TA . Lipid‐binding proteins: a family of fatty acid and retinoid 796 transport proteins. Adv Protein Chem. 1994;45:89–151. 8154375 10.1016/s0065-3233(08)60639-7

[pro70608-bib-0004] Barnes CA , Robertson AJ , Louis JM , Anfinrud P , Bax A . Observation of β‐amyloid peptide oligomerization by pressure‐jump NMR spectroscopy. J Am Chem Soc. 2019;141:13762–13766. 31432672 10.1021/jacs.9b06970 PMC9357264

[pro70608-bib-0005] Basehore HK , Ropson IJ . Residual interactions in unfolded bile acid‐binding protein by 19F NMR. Protein Sci. 2011;20:327–335. 21280124 10.1002/pro.563 PMC3048417

[pro70608-bib-0006] Berezovsky IN , Kirzhner VM , Kirzhner A , Trifonov EN . Protein folding: looping from hydrophobic nuclei. Proteins. 2001;45:346–350. 11746682 10.1002/prot.1155

[pro70608-bib-0007] Bergasa‐Caceres F , Haas E , Rabitz HA . Nature's shortcut to protein folding. J Phys Chem B. 2019;123:4463–4476. 30901220 10.1021/acs.jpcb.8b11634

[pro70608-bib-0008] Berner F , Kovermann M . Including the ensemble of unstructured conformations in the analysis of protein's native state by high‐pressure NMR spectroscopy. Angew Chem Int Ed Engl. 2024;63:e202401343. 38656763 10.1002/anie.202401343

[pro70608-bib-0009] Bintee B , Banerjee R , Hegde M , Vishwa R , Alqahtani MS , Abbas M , et al. Exploring bile acid transporters as key players in cancer development and treatment: evidence from preclinical and clinical studies. Cancer Lett. 2025;609:217324. 39571783 10.1016/j.canlet.2024.217324

[pro70608-bib-0010] Borgström B , Barrowman JA , Lindström M . Roles of bile acids in intestinal lipid digestion and absorption. In: Danielsson H , Sjövall J , editors. Sterols and bile acids. Volume 12. Amsterdam, The Netherlands: Elsevier; 1985. p. 405–425.

[pro70608-bib-0011] Budyak IL , Krishnan B , Marcelino‐Cruz AM , Ferrolino MC , Zhuravleva A , Gierasch LM . Early folding events protect aggregation‐prone regions of a β‐rich protein. Structure. 2013;21:476–485. 23454187 10.1016/j.str.2013.01.013 PMC3630246

[pro70608-bib-0012] Burns LL , Dalessio PM , Ropson IJ . Folding mechanism of three structurally similar beta‐sheet proteins. Proteins. 1998;33:107–118. 9741849 10.1002/(sici)1097-0134(19981001)33:1<107::aid-prot10>3.0.co;2-p

[pro70608-bib-0013] Burns LL , Ropson IJ . Folding of intracellular retinol and retinoic acid binding proteins. Proteins. 2001;43:292–302. 11288179 10.1002/prot.1040

[pro70608-bib-0014] Caro JA , Wand AJ . Practical aspects of high‐pressure NMR spectroscopy and its applications in protein biophysics and structural biology. Methods. 2018;148:67–80. 29964175 10.1016/j.ymeth.2018.06.012 PMC6133745

[pro70608-bib-0015] Chattopadhyay K , Zhong S , Yeh SR , Rousseau DL , Frieden C . The intestinal fatty acid binding protein: the role of turns in fast and slow folding processes. Biochemistry. 2002;41:4040–4047. 11900547 10.1021/bi012042l

[pro70608-bib-0016] Chen CR , Makhatadze GI . Molecular determinant of the effects of hydrostatic pressure on protein folding stability. Nat Commun. 2017;8:14561. 28169271 10.1038/ncomms14561 PMC5309723

[pro70608-bib-0017] Chiang JYL , Ferrell JM . Bile acid receptors FXR and TGR5 signaling in fatty liver diseases and therapy. Am J Physiol Gastrointest Liver Physiol. 2020;318:G554–G573. 31984784 10.1152/ajpgi.00223.2019 PMC7099488

[pro70608-bib-0018] Chuang S , Velkov T , Horne J , Porter CJ , Scanlon MJ . Characterization of the drug binding specificity of rat liver fatty acid binding protein. J Med Chem. 2008;51:3755–3764. 18533710 10.1021/jm701192w

[pro70608-bib-0019] Cistola DP , Kim K , Rogl H , Frieden C . Fatty acid interactions with a helix‐less variant of intestinal fatty acid‐binding protein. Biochemistry. 1996;35:7559–7565. 8652536 10.1021/bi952912x

[pro70608-bib-0020] Cogliati C , Ragona L , D'Onofrio M , Günther U , Whittaker S , Ludwig C , et al. Site‐specific investigation of the steady‐state kinetics and dynamics of the multistep binding of bile acid molecules to a lipid carrier protein. Chem Eur J. 2010;16:11300–11310. 20715194 10.1002/chem.201000498

[pro70608-bib-0021] Cooper A . Thermodynamic fluctuations in protein molecules. Proc Natl Acad Sci USA. 1976;73:2740–2741. 1066687 10.1073/pnas.73.8.2740 PMC430724

[pro70608-bib-0022] Corsico B , Cistola DP , Frieden C , Storch J . The helical domain of intestinal fatty acid binding protein is critical for collisional transfer of fatty acids to phospholipid membranes. Proc Natl Acad Sci USA. 1998;95:12174–12178. 9770459 10.1073/pnas.95.21.12174 PMC22804

[pro70608-bib-0023] Dalessio PM , Fromholt SE , Ropson IJ . The role of Trp‐82 in the folding of intestinal fatty acid binding protein. Proteins. 2005;61:176–183. 10.1002/prot.20463 16080148

[pro70608-bib-0024] Delassio PM , Ropson IJ . Beta‐sheet proteins with nearly identical structures have different folding intermediates. Biochemistry. 2000;39:860–871. 10653629 10.1021/bi991937j

[pro70608-bib-0025] Ding L , Yang L , Wang Z , Huang W . Bile acid nuclear receptor FXR and digestive system diseases. Acta Pharm Sin B. 2015;5:135–144. 26579439 10.1016/j.apsb.2015.01.004 PMC4629217

[pro70608-bib-0026] D'Onofrio M , Gianolio E , Ceccon A , Arena F , Zanzoni S , Fushman D , et al. High relaxivity supramolecular adducts between human‐liver fatty‐acid‐binding protein and amphiphilic Gd(III) complexes: structural basis for the design of intracellular targeting MRI probes. Chemistry. 2012;18:9919–9928. 22763949 10.1002/chem.201103778

[pro70608-bib-0027] D'Onofrio M , Ragona L , Fessas D , Signorelli M , Ugolini R , Pedò M , et al. NMR unfolding studies on a liver bile acid binding protein reveal a global two‐state unfolding and localized singular behaviors. Arch Biochem Biophys. 2009;481:21–29. 18977333 10.1016/j.abb.2008.10.017

[pro70608-bib-0028] Dubois C , Herrada I , Barthe P , Roumestand C . Combining high‐pressure perturbation with NMR spectroscopy for a structural and dynamical characterization of protein folding pathways. Molecules. 2020;25:5551. 33256081 10.3390/molecules25235551 PMC7731413

[pro70608-bib-0029] Eliseo T , Ragona L , Catalano M , Assfalg M , Paci M , Zetta L , et al. Structural and dynamic determinants of ligand binding in the ternary complex of chicken liver bile acid binding protein with two bile salts revealed by NMR. Biochemistry. 2007;46:12557–12567. 17929837 10.1021/bi7013085

[pro70608-bib-0030] Fossat MJ , Dao TP , Jenkins K , Dellarole M , Yang Y , McCallum SA , et al. High‐resolution mapping of a repeat protein folding free energy landscape. Biophys J. 2016;111:2368–2376. 27926838 10.1016/j.bpj.2016.08.027 PMC5153537

[pro70608-bib-0031] Franzoni L , Lücke C , Pérez C , Cavazzini D , Rademacher M , Ludwig C , et al. Structure and backbone dynamics of apo‐ and holo‐cellular retinol‐binding protein in solution. J Biol Chem. 2002;277:21983–21997. 11934897 10.1074/jbc.M201994200

[pro70608-bib-0032] Girard E , Lopes P , Spoerner M , Dhaussy AC , Prangé T , Kalbitzer HR , et al. Equilibria between conformational states of the Ras oncogene protein revealed by high pressure crystallography. Chem Sci. 2022;13:2001–2010. 35308861 10.1039/d1sc05488k PMC8848853

[pro70608-bib-0033] Glatz JFC , Veerkamp JH . A radiochemical procedure for the assay of fatty acid binding by proteins. Anal Biochem. 1983;132:89–95. 6194713 10.1016/0003-2697(83)90429-3

[pro70608-bib-0034] González JM , Fisher SZ . Structural analysis of ibuprofen binding to human adipocyte fatty‐acid binding protein (FABP4). Acta Crystallogr F Struct Biol Commun. 2015;71(Pt 2):163–170. 25664790 10.1107/S2053230X14027897 PMC4321470

[pro70608-bib-0035] Herr FM , Matarese V , Bernlohr DA , Storch J . Surface lysine residues modulate the collisional transfer of fatty acid from adipocyte fatty acid binding protein to membranes. Biochemistry. 1995;34:11840–11845. 7547918 10.1021/bi00037a023

[pro70608-bib-0036] Herrada I , Barthe P , Vanheusden M , DeGuillen K , Mammri L , Delbecq S , et al. Monitoring unfolding of Titin I27 single and bi domain with high‐pressure NMR spectroscopy. Biophys J. 2018;115:341–352. 30021109 10.1016/j.bpj.2018.06.010 PMC6051020

[pro70608-bib-0037] Hodsdon ME , Cistola DP . Discrete backbone disorder in the nuclear magnetic resonance structure of apo intestinal fatty acid‐binding protein: implications for the mechanism of ligand entry. Biochemistry. 1997a;36:1450–1460. 9063893 10.1021/bi961890r

[pro70608-bib-0038] Hodsdon ME , Cistola DP . Ligand binding alters the backbone mobility of intestinal fatty acid‐binding protein as monitored by 15N NMR relaxation and 1H exchange. Biochemistry. 1997b;36:2278–2290. 9047330 10.1021/bi962018l

[pro70608-bib-0039] Hodsdon ME , Frieden C . Intestinal fatty acid binding protein: the folding mechanism as determined by NMR studies. Biochemistry. 2001;40:732–742. 11170390 10.1021/bi001518i

[pro70608-bib-0040] Hofmann AF . The enterohepatic circulation of bile acids in mammals: form and functions. Front Biosci (Landmark Ed). 2009;14:2584–2598. 19273221 10.2741/3399

[pro70608-bib-0041] Horváth G , Balterer B , Micsonai A , Kardos J , Toke O . Multiple timescale dynamic analysis of functionally‐impairing mutations in human ileal bile acid‐binding protein. Int J Mol Sci. 2022;23:11346. 36232642 10.3390/ijms231911346 PMC9569817

[pro70608-bib-0042] Horváth G , Bencsura Á , Simon Á , Tochtrop GP , DeKoster GT , Covey DF , et al. Structural determinants of ligand binding in the ternary complex of human ileal bile acid binding protein with glycocholate and glycochenodeoxycholate obtained from solution NMR. FEBS J. 2016;283:541–555. 26613247 10.1111/febs.13610

[pro70608-bib-0043] Horváth G , Biczók L , Majer Z , Kovács M , Micsonai A , Kardos J , et al. Structural insight into a partially unfolded state preceding aggregation in an intracellular lipid‐binding protein. FEBS J. 2017;284:3637–3661. 28886229 10.1111/febs.14264

[pro70608-bib-0044] Horváth G , Egyed O , Tang C , Kovács M , Micsonai A , Kardos J , et al. Ligand entry in human ileal bile acid‐binding protein is mediated by histidine protonation. Sci Rep. 2019;9:4825. 30886237 10.1038/s41598-019-41180-7 PMC6423008

[pro70608-bib-0045] Horváth G , Egyed O , Toke O . Temperature dependence of backbone dynamics in human ileal bile acid‐binding protein: implications for the mechanism of ligand binding. Biochemistry. 2014;53:5186–5198. 25073073 10.1021/bi500553f

[pro70608-bib-0046] Horváth G , Király P , Tárkányi G , Toke O . Internal motions and exchange processes in human ileal bile acid binding protein as studied by backbone 15N nuclear magnetic resonance spectroscopy. Biochemistry. 2012;51:1848–1861. Erratum in Biochemistry. 51:10119. 22329738 10.1021/bi201588q

[pro70608-bib-0047] Houten SM , Watanabe M , Auwerx J . Endocrine functions of bile acids. EMBO J. 2006;25:1419–1425. 16541101 10.1038/sj.emboj.7601049 PMC1440314

[pro70608-bib-0048] Kalbitzer HR , Rosnizeck IC , Munte CE , Narayanan SP , Kropf V , Spoerner M . Intrinsic allosteric inhibition of signaling proteins by targeting rare interaction states detected by high‐pressure NMR spectroscopy. Angew Chem Int Ed Engl. 2013;52:14242–14246. 24218090 10.1002/anie.201305741

[pro70608-bib-0049] Kay LE , Keifer P , Saarinen T . Pure absorption gradient enhanced heteronuclear single quantum correlation spectroscopy with improved sensitivity. J Am Chem Soc. 1992;114:10663–10665.

[pro70608-bib-0050] Kim HK , Storch J . Mechanism of free fatty acid transfer from rat heart fatty acid‐binding protein to phospholipid membranes. Evidence for a collisional process. J Biol Chem. 1992;267:20051–20056. 1400322

[pro70608-bib-0051] Kim K , Cistola DP , Frieden C . Intestinal fatty acid‐binding protein: the structure and stability of a helix‐less variant. Biochemistry. 1996;35:7553–7558. 8652535 10.1021/bi9529115

[pro70608-bib-0052] Kim K , Frieden C . Turn scanning by site‐directed mutagenesis: application to the protein folding problem using the intestinal fatty acid binding protein. Protein Sci. 1998;7:1821–1828. 10082380 10.1002/pro.5560070818 PMC2144079

[pro70608-bib-0053] Kim K , Ramanathan R , Frieden C . Intestinal fatty acid binding protein: a specific residue in one turn appears to stabilize the native structure and be responsible for slow refolding. Protein Sci. 1997;6:364–372. 9041638 10.1002/pro.5560060212 PMC2143660

[pro70608-bib-0054] Kitahara R , Akasaka K . Close identity of a pressure‐stabilized intermediate with a kinetic intermediate in protein folding. Proc Natl Acad Sci USA. 2003;100:3167–3172. 12629216 10.1073/pnas.0630309100 PMC152264

[pro70608-bib-0055] Kitahara R , Simorellis AK , Hata K , Maeno A , Yokoyama S , Koide S , et al. A delicate interplay of structure, dynamics, and thermodynamics for function: a high pressure NMR study of outer surface protein a. Biophys J. 2012;102:916–926. 22385863 10.1016/j.bpj.2011.12.010 PMC3283806

[pro70608-bib-0056] Kleckner IR , Foster MP . GUARDD: user‐friendly MATLAB software for rigorous analysis of CPMG RD NMR data. J Biomol NMR. 2012;52:11–22. 22160811 10.1007/s10858-011-9589-y PMC3593345

[pro70608-bib-0057] Korzhnev DM , Kloiber K , Kay LE . Multiple‐quantum relaxation dispersion NMR spectroscopy probing millisecond time‐scale dynamics in proteins: theory and application. J Am Chem Soc. 2004;126:7320–7329. 15186169 10.1021/ja049968b

[pro70608-bib-0058] Kouvatsos N , Meldrum JK , Searle MS , Thomas NR . Coupling ligand recognition to protein folding in an engineered variant of rabbit ileal lipid binding protein. Chem Commun (Camb). 2006;44:4623–4625. 10.1039/b610130e 17082863

[pro70608-bib-0059] Kramer W , Girbig F , Gutjahr U , Kowalewski S , Jouvenal K , Müller G , et al. Intestinal bile acid absorption. Na(+)‐dependent bile acid transport activity in rabbit small intestine correlates with the coexpression of an integral 93‐kDa and a peripheral 14‐kDa bile acid‐binding membrane protein along the duodenum‐ileum axis. J Biol Chem. 1993;268:18035–18046. 8349683

[pro70608-bib-0060] Kurz M , Brachvogel V , Matter H , Stengelin S , Thüring H , Kramer W . Insights into the bile acid transportation system: the human ileal lipid‐binding protein‐cholyltaurine complex and its comparison with homologous structures. Proteins. 2003;50:312–328. 12486725 10.1002/prot.10289

[pro70608-bib-0061] Lefebvre P , Cariou B , Lien F , Kuipers F , Staels B . Role of bile acids and bile acid receptors in metabolic regulation. Physiol Rev. 2009;89:147–191. 19126757 10.1152/physrev.00010.2008

[pro70608-bib-0062] Li E , Locke B , Yang NC , Ong DE , Gordon JI . Characterization of rat cellular retinol‐binding protein II expressed in *Escherichia coli* . J Biol Chem. 1987;262:13773–13779. 3308883

[pro70608-bib-0063] Li H , Frieden C . Phenylalanine side chain behavior of the intestinal fatty acid‐binding protein: the effect of urea on backbone and side chain stability. J Biol Chem. 2005a;280:38556–38561. 16162507 10.1074/jbc.M505435200

[pro70608-bib-0064] Li H , Frieden C . NMR studies of 4‐19F‐phenylalanine‐labeled intestinal fatty acid binding protein: evidence for conformational heterogeneity in the native state. Biochemistry. 2005b;44:2369–2377. 15709749 10.1021/bi047600l

[pro70608-bib-0065] Li H , Frieden C . Observation of sequential steps in the folding of intestinal fatty acid binding protein using a slow folding mutant and 19F NMR. Proc Natl Acad Sci USA. 2007a;104:11993–11998. 17615232 10.1073/pnas.0705253104 PMC1924545

[pro70608-bib-0066] Li H , Frieden C . Comparison of C40/82A and P27A C40/82A barstar mutants using 19F NMR. Biochemistry. 2007b;46:4337–4347. 17371049 10.1021/bi6026083

[pro70608-bib-0067] Li H , Yamada H , Akasaka K . Effect of pressure on individual hydrogen bonds in proteins. Basic pancreatic trypsin inhibitor. Biochemistry. 1998;37:1167–1173. 9477939 10.1021/bi972288j

[pro70608-bib-0068] Loria JP , Rance M , Palmer AG III . A relaxation‐compensated Carr‐Purcell‐Meiboom‐Gill sequence for characterizing chemical exchange by NMR spectroscopy. J Am Chem Soc. 1999;121:2331–2332.

[pro70608-bib-0069] Lu J , Cistola DP , Li E . Two homologous rat cellular retinol‐binding proteins differ in local conformational flexibility. J Mol Biol. 2003;330:799–812. 12850148 10.1016/s0022-2836(03)00629-6

[pro70608-bib-0070] Makishima M , Okamoto AY , Repa JJ , Tu H , Learned RM , Luk A , et al. Identification of a nuclear receptor for bile acids. Science. 1999;284:1362–1365. 10334992 10.1126/science.284.5418.1362

[pro70608-bib-0071] Markley JL , Bax A , Arata Y , Hilbers CW , Kaptein R , Sykes BD , et al. Recommendations for the presentation of NMR structures of proteins and nucleic acids. Pure Appl Chem. 1998;70:117–142. 10.1006/jmbi.1998.1852 9671561

[pro70608-bib-0072] Menozzi I , Polverini E , Berni R . Deciphering protein dynamics changes along the pathway of retinol by cellular retinol‐binding proteins 1 and 2. Arch Biochem Biphys. 2018;645:107–116. 10.1016/j.abb.2018.03.020 29567208

[pro70608-bib-0073] Mulder FA , Skrynnikov NR , Hon B , Dahlquist FW , Kay LE . Measurement of slow (micros‐ms) time scale dynamics in protein side chains by (15)N relaxation dispersion NMR spectroscopy: application to Asn and Gln residues in a cavity mutant of T4 lysozyme. J Am Chem Soc. 2001;123:967–975. 11456632 10.1021/ja003447g

[pro70608-bib-0074] Munte CE , Beck Erlach M , Kremer W , Koehler J , Kalbitzer HR . Distinct conformational states of the Alzheimer β‐amyloid peptide can be detected by high‐pressure NMR spectroscopy. Angew Chem Int Ed Engl. 2013;52:8943–8947. 23843225 10.1002/anie.201301537

[pro70608-bib-0075] Nakahara M , Furuya N , Takagaki K , Sugaya T , Hirota K , Fukamizu A , et al. Ileal bile acid‐binding protein, functionally associated with the farnesoid X receptor or the ileal bile acid transporter, regulates bile acid activity in the small intestine. J Biol Chem. 2005;280:42283–42289. 16230354 10.1074/jbc.M507454200

[pro70608-bib-0076] Nikiforovich GV , Frieden C . The search for local native‐like nucleation centers in the unfolded state of beta ‐sheet proteins. Proc Natl Acad Sci USA. 2002;99:10388–10393. 12140369 10.1073/pnas.162362199 PMC124924

[pro70608-bib-0077] Pace CN , Vajdos F , Fee L , Grimsley G , Gray T . How to measure and predict the molar absorption coefficient of a protein. Protein Sci. 1995;4:2411–2423. 8563639 10.1002/pro.5560041120 PMC2143013

[pro70608-bib-0078] Parks DJ , Blanchard SG , Bledsoe RK , Chandra G , Consler TG , Kliewer SA , et al. Bile acids: natural ligands for an orphan nuclear receptor. Science. 1999;284:1365–1368. 10334993 10.1126/science.284.5418.1365

[pro70608-bib-0079] Pastore A , Temussi PA . The protein unfolded state: one, no one and one hundred thousand. J Am Chem Soc. 2022;144:22352–22357. 36450361 10.1021/jacs.2c07696 PMC9756289

[pro70608-bib-0080] Patil R , Laguerre A , Wielens J , Headey SJ , Williams ML , Hughes ML , et al. Characterization of two distinct modes of drug binding to human intestinal fatty acid binding protein. ACS Chem Biol. 2014;9:2526–2534. 25144524 10.1021/cb5005178

[pro70608-bib-0081] Pedò M , Löhr F , D'Onofrio M , Assfalg M , Dötsch V , Molinari H . NMR studies reveal the role of biomembranes in modulating ligand binding and release by intracellular bile acid binding proteins. J Mol Biol. 2009;394:852–863. 19836400 10.1016/j.jmb.2009.10.014

[pro70608-bib-0082] Perino A , Demagny H , Velazquez‐Villegas L , Schoonjans K . Molecular physiology of bile acid signaling in health, disease, and aging. Physiol Rev. 2021;101:683–731. 32790577 10.1152/physrev.00049.2019

[pro70608-bib-0083] Pettersen EF , Goddard TD , Huang CC , Couch GS , Greenblatt DM , Meng EC , et al. UCSF chimera—a visualization system for exploratory research and analysis. J Comput Chem. 2004;25:1605–1612. 15264254 10.1002/jcc.20084

[pro70608-bib-0084] Ragona L , Catalano M , Luppi M , Cicero D , Eliseo T , Foote J , et al. NMR dynamic studies suggest that allosteric activation regulates ligand binding in chicken liver bile acid‐binding protein. J Biol Chem. 2006;281:9697–9709. 16439356 10.1074/jbc.M513003200

[pro70608-bib-0085] Ragona L , Pagano K , Tomaselli S , Favretto F , Ceccon A , Zanzoni S , et al. The role of dynamics in modulating ligand exchange in intracellular lipid binding proteins. Biochim Biophys Acta, Proteins Proteomics. 2014;1844:1268–1278. 10.1016/j.bbapap.2014.04.011 24768771

[pro70608-bib-0086] Roche J , Caro JA , Norberto DR , Barthe P , Roumestand C , Schlessman JL , et al. Cavities determine the pressure unfolding of proteins. Proc Natl Acad Sci USA. 2012;109:6945–6950. 22496593 10.1073/pnas.1200915109 PMC3344970

[pro70608-bib-0087] Roche J , Royer CA , Roumestand C . Monitoring protein folding through high pressure NMR spectroscopy. Prog Nucl Magn Reson Spectrosc. 2017;102‐103:15–31. 10.1016/j.pnmrs.2017.05.003 29157491

[pro70608-bib-0088] Ropson IJ , Boyer JA , Dalessio PM . A residual structure in unfolded intestinal fatty acid binding protein consists of amino acids that are neighbors in the native state. Biochemistry. 2006;45:2608–2617. 16489754 10.1021/bi052091o

[pro70608-bib-0089] Ropson IJ , Boyer JA , Schaeffer BA , Dalessio PM . Comparison of the folding mechanism of highly homologous proteins in the lipid‐binding protein family. Proteins. 2009;75:799–806. 19003989 10.1002/prot.22286 PMC6336386

[pro70608-bib-0090] Ropson IJ , Frieden C . Dynamic NMR spectral analysis and protein folding: identification of a highly populated folding intermediate of rat intestinal fatty acid‐binding protein by 19F NMR. Proc Natl Acad Sci USA. 1992;89:7222–7226. 1496015 10.1073/pnas.89.15.7222 PMC49678

[pro70608-bib-0091] Rose GD . From propensities to patterns to principles in protein folding. Proteins. 2025;93:105–111. 37353953 10.1002/prot.26540

[pro70608-bib-0092] Rosenman DJ , Clemente N , Ali M , García AE , Wang C . High pressure NMR reveals conformational perturbations by disease‐causing mutations in amyloid β‐peptide. Chem Commun (Camb). 2018;54:4609–4612. 29670961 10.1039/c8cc01674g

[pro70608-bib-0093] Roumestand C , Dudas E , Puglisi R , Calió A , Barthe P , Temussi PA , et al. Understanding the relationship between pressure and temperature unfolding of proteins. JACS Au. 2025;5:1940–1955. 40313814 10.1021/jacsau.5c00185 PMC12042054

[pro70608-bib-0094] Schroer MA , Paulus M , Jeworrek C , Krywka C , Schmacke S , Zhai Y , et al. High‐pressure SAXS study of folded and unfolded ensembles of proteins. Biophys J. 2010;99:3430–3437. 21081092 10.1016/j.bpj.2010.09.046 PMC2980736

[pro70608-bib-0095] Scott K , Keeler J , Van QN , Shaka AJ . One‐dimensional NOE experiments using pulsed field gradients. J Magn Reson. 1997;125:302–324.

[pro70608-bib-0096] Sessler RJ , Noy N . A ligand‐activated nuclear localization signal in cellular retinoic acid binding protein‐II. Mol Cell. 2005;18:343–353. 15866176 10.1016/j.molcel.2005.03.026

[pro70608-bib-0097] Small DM , Dowling RH , Redinger RN . The enterohepatic circulation of bile salts. Arch Intern Med. 1972;130:552–573. 4627839

[pro70608-bib-0098] Smeller L . Pressure‐temperature phase diagrams of biomolecules. Biochim Biophys Acta. 2002;1595:11–29. 11983384 10.1016/s0167-4838(01)00332-6

[pro70608-bib-0099] Steele RA , Emmert DA , Kao J , Hodsdon ME , Frieden C , Cistola DP . The three‐dimensional structure of a helix‐less variant of intestinal fatty acid‐binding protein. Protein Sci. 1998;7:1332–1339. 9655337 10.1002/pro.5560070609 PMC2144039

[pro70608-bib-0100] Teski T , Horváth G , Toke O . Determinants of site‐selectivity in human ileal bile acid‐binding protein by NMR dynamic analysis of a functionally‐impaired mutant. J Struct Biol. 2025;217:108202. 40268173 10.1016/j.jsb.2025.108202

[pro70608-bib-0101] Thakur AK , Meng W , Gierasch LM . Local and non‐local topological information in the denatured state ensemble of a β‐barrel protein. Protein Sci. 2018;27:2062–2072. 30252171 10.1002/pro.3516 PMC6237703

[pro70608-bib-0102] Tochtrop GP , Bruns JL , Tang C , Covey DF , Cistola DP . Steroid ring hydroxylation patterns govern cooperativity in human bile acid binding protein. Biochemistry. 2003;42:11561–11567. 14529265 10.1021/bi0346502

[pro70608-bib-0103] Tochtrop GP , DeKoster GT , Covey DF , Cistola DP . A single hydroxyl group governs ligand site selectivity in human ileal bile acid binding protein. J Am Chem Soc. 2004;126:11024–11029. 15339188 10.1021/ja047589c

[pro70608-bib-0104] Tochtrop GP , Richter K , Tang C , Toner JJ , Covey DF , Cistola DP . Energetics by NMR: site‐specific binding in a positively cooperative system. Proc Natl Acad Sci USA. 2002;99:1847–1852. 11854486 10.1073/pnas.012379199 PMC122282

[pro70608-bib-0105] Toke O . Structural and dynamic determinants of molecular recognition in bile acid‐binding proteins. Int J Mol Sci. 2022;23:505. 35008930 10.3390/ijms23010505 PMC8745080

[pro70608-bib-0106] Toke O , Monsey JD , Cistola DP . Kinetic mechanism of ligand binding in human ileal bile acid binding protein as determined by stopped‐flow fluorescence analysis. Biochemistry. 2007;46:5427–5436. 17432832 10.1021/bi700030r

[pro70608-bib-0107] Toke O , Monsey JD , DeKoster GT , Tochtrop GP , Tang C , Cistola DP . Determinants of cooperativity and site‐selectivity in human ileal bile acid‐binding protein. Biochemistry. 2006;45:727–737. 16411748 10.1021/bi051781p

[pro70608-bib-0108] Tollinger M , Skrynnikov NR , Mulder FA , Forman‐Kay JD , Kay LE . Slow dynamics of folded and unfolded states of an SH3 domain. J Am Chem Soc. 2001;123:11341–11352. 11707108 10.1021/ja011300z

[pro70608-bib-0109] Tomaselli S , Zanzoni S , Ragona L , Gianolio E , Aime S , Assfalg M , et al. Solution structure of the supramolecular adduct between a liver cytosolic bile acid binding protein and a bile acid‐based gadolinium(III)‐chelate, a potential hepatospecific magnetic resonance imaging contrast agent. J Med Chem. 2008;51:6782–67092. 18939814 10.1021/jm800820b

[pro70608-bib-0110] Veerkamp JH , Maatman RG . Cytoplasmic fatty acid‐binding proteins: their structure and genes. Prog Lipid Res. 1995;34:17–52. 7644552 10.1016/0163-7827(94)00005-7

[pro70608-bib-0111] Vemulapalli SPB , Becker S , Griesinger C , Rezaei‐Ghaleh N . Combined high‐pressure and multiquantum NMR and molecular simulation propose a role for N‐terminal salt bridges in amyloid‐beta. J Phys Chem Lett. 2021;12:9933–9939. 34617758 10.1021/acs.jpclett.1c02595 PMC8521524

[pro70608-bib-0112] Vidugiris GJ , Truckses DM , Markley JL , Royer CA . High‐pressure denaturation of staphylococcal nuclease proline‐to‐glycine substitution mutants. Biochemistry. 1996;35:3857–3864. 8620010 10.1021/bi952012g

[pro70608-bib-0113] Williamson MP , Akasaka K , Refaee M . The solution structure of bovine pancreatic trypsin inhibitor at high pressure. Protein Sci. 2003;12:1971–1979. 12930996 10.1110/ps.0242103 PMC2323994

[pro70608-bib-0114] Yeh SR , Ropson IJ , Rousseau DL . Hierarchical folding of intestinal fatty acid binding protein. Biochemistry. 2001;40:4205–4210. 11284675 10.1021/bi0155044

