# Supplementary material for: Functionally related internal fluctuations in human ileal bile acid‐binding protein by high pressure nuclear magnetic resonance
Source: Protein Sci. 2026 May 4;35(6):e70608. doi: 10.1002/pro.70608 (PMC13137299; doi:10.1002/pro.70608)
Supplement: Supplementary file 1 — Figure S1. Reversibility of hI‐BABP pressure denaturation. (a) 1H‐15N HSQC NMR spectra of 15N‐labeled hI‐BABP (0.4 mM, 18°C, pH = 6.3) at atmospheric pressure recorded before (black) and after (cyan) the measurement series in the 1–3000 bar pressure range. The spectrum in cyan is shifted upward for better viewing. The spectrum in gray was collected at 3000 bar. (b) One‐dimensional 1H‐NMR spectra of the same sample showing the full 1H spectral range at atmospheric pressure before (black) and after (cyan) the completion of the pressurization series. Figure S2. Reversibility of hI‐BABP pressure denaturation. (a) 1H and (b) 15N chemical shift differences as a function of the amino acid sequence as determind from 1H‐15N HSQC NMR spectra of 15N‐labeled hI‐BABP (Figure S1) recorded at atmospheric pressure before and after the measurement series in the 1–3000 bar pressure range. (c) Ratio of peak intensities as a function of amino acid sequence between the two measurements at 1 bar. Figure S3. (a) Sequence alignment of human I‐BABP and rat I‐FABP discussed in the main text. Residue numbering is according to hI‐BABP. Secondary structural elements indicated at the top are according to apo hI‐BABP. The break occurring in βF is indicated in gray. (b) Conserved pairwise interactions in human I‐BABP (PDB: 1O1U; Kurz et al., 2003, left) and rat I‐FABP (PDB: 1AEL; Hodsdon & Cistola, 1997a, right). Short‐ and long‐range interactions are depicted in dashed and solid lines, respectively. Residues involved in short‐range, long‐range, or both short‐ and long‐range interactions are depicted in yellow, blue, and magenta, respectively. Residues involved in multiple long‐range interactions are marked with a green dot. Note the missing residues in panel (a) affecting the numbering in panel (b). Table S1. “Local” thermodynamic parameters of pressure‐induced unfolding obtained for apo hI‐BABP in 20 mM K‐phosphate, 50 mM KCl, 0.05% NaN3, pH = 6.3 at 291 K. Table S2. Exchange parameters derived [file PRO-35-e70608-s001.docx]

**SUPPLEMENTARY MATERIAL**

**Functionally-related Internal Fluctuations in Human Ileal Bile Acid-Binding Protein**

**by High Pressure NMR**

Tamara Teski^1,2^, Bence Balterer^1^, Gergő Horváth^1^, Gábor Turczel^1^, and Orsolya Toke^1*^

*^1^ Centre for Structural Science, HUN-REN Research Centre for Natural Sciences, 2 Magyar tudósok körútja, Budapest H-1117, Hungary*

*^2^ Doctoral School of Biology, Institute of Biology, ELTE Eötvös Loránd University, Budapest 1117, Hungary*


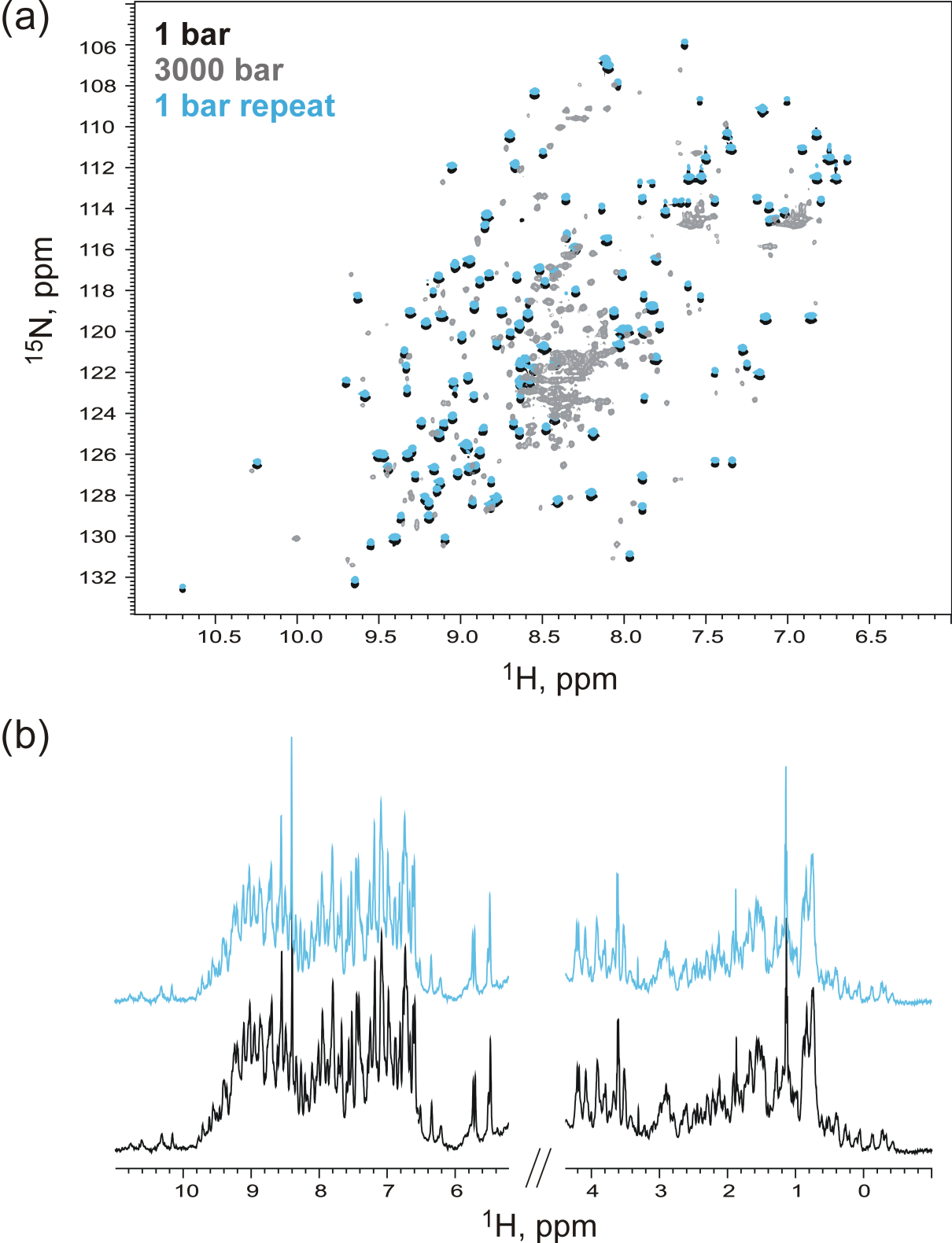


**Figure S1.** Reversibility of hI-BABP pressure denaturation. (a) ^1^H-^15^N HSQC NMR spectra of ^15^N-labeled hI-BABP (0.4 mM, 18 °C, pH=6.3) at atmospheric pressure recorded before (black) and after (cyan) the measurement series in the 1-3000 bar pressure range. The spectrum in cyan is shifted upward for better viewing. The spectrum in grey was collected at 3000 bar. (b) One-dimensional ^1^H-NMR spectra of the same sample showing the full ^1^H spectral range at atmospheric pressure before (black) and after (cyan) the completion of the pressurization series.


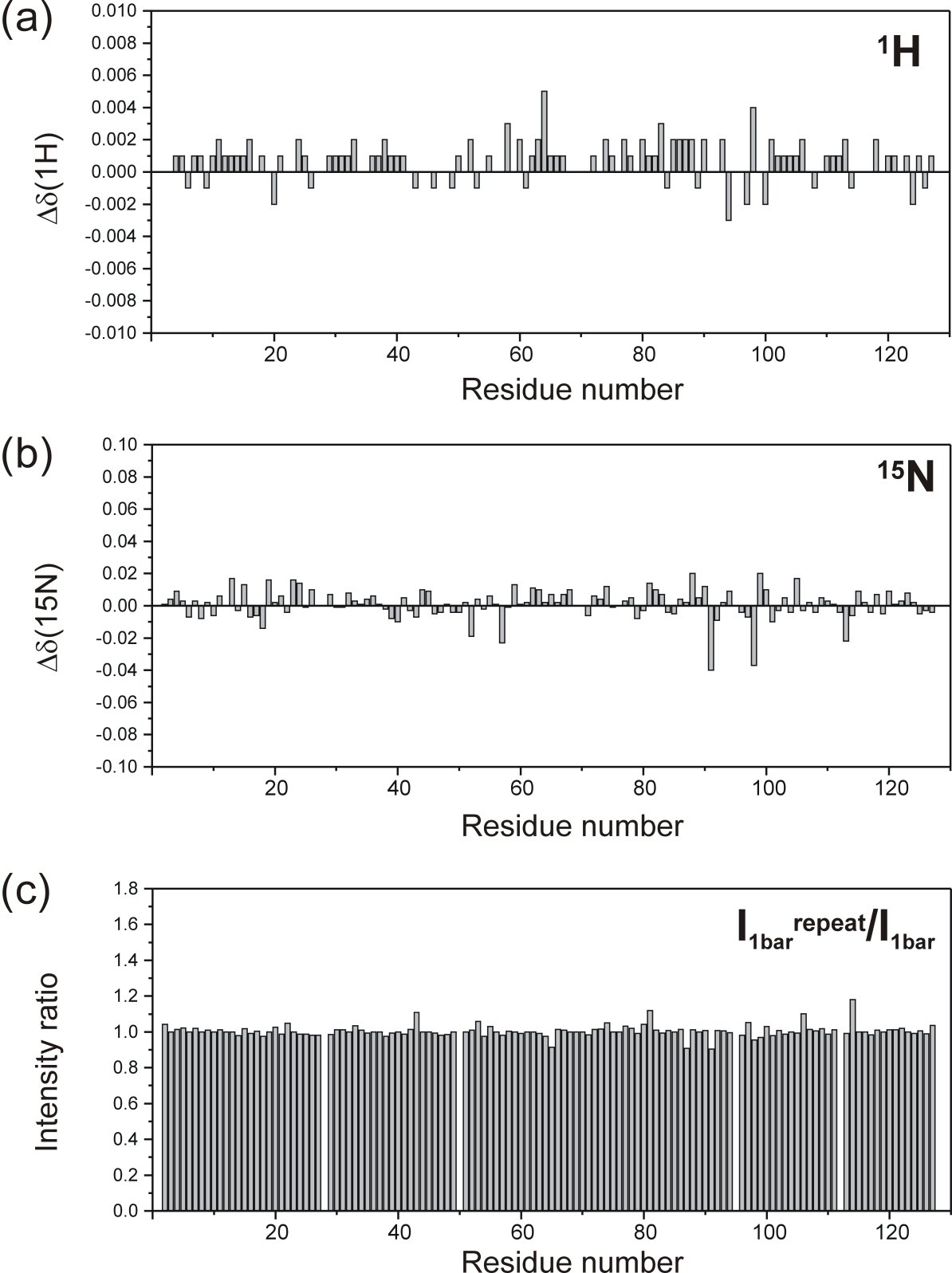


**Figure S2.** Reversibility of hI-BABP pressure denaturation. (a) ^1^H and (b) ^15^N chemical shift differences as a function of the amino acid sequence as determind from ^1^H-^15^N HSQC NMR spectra of ^15^N-labeled hI-BABP (Figure S1) recorded at atmospheric pressure before and after the measurement series in the 1-3000 bar pressure range. (c) Ratio of peak intensities as a function of amino acid sequence between the two measurements at 1 bar.

*
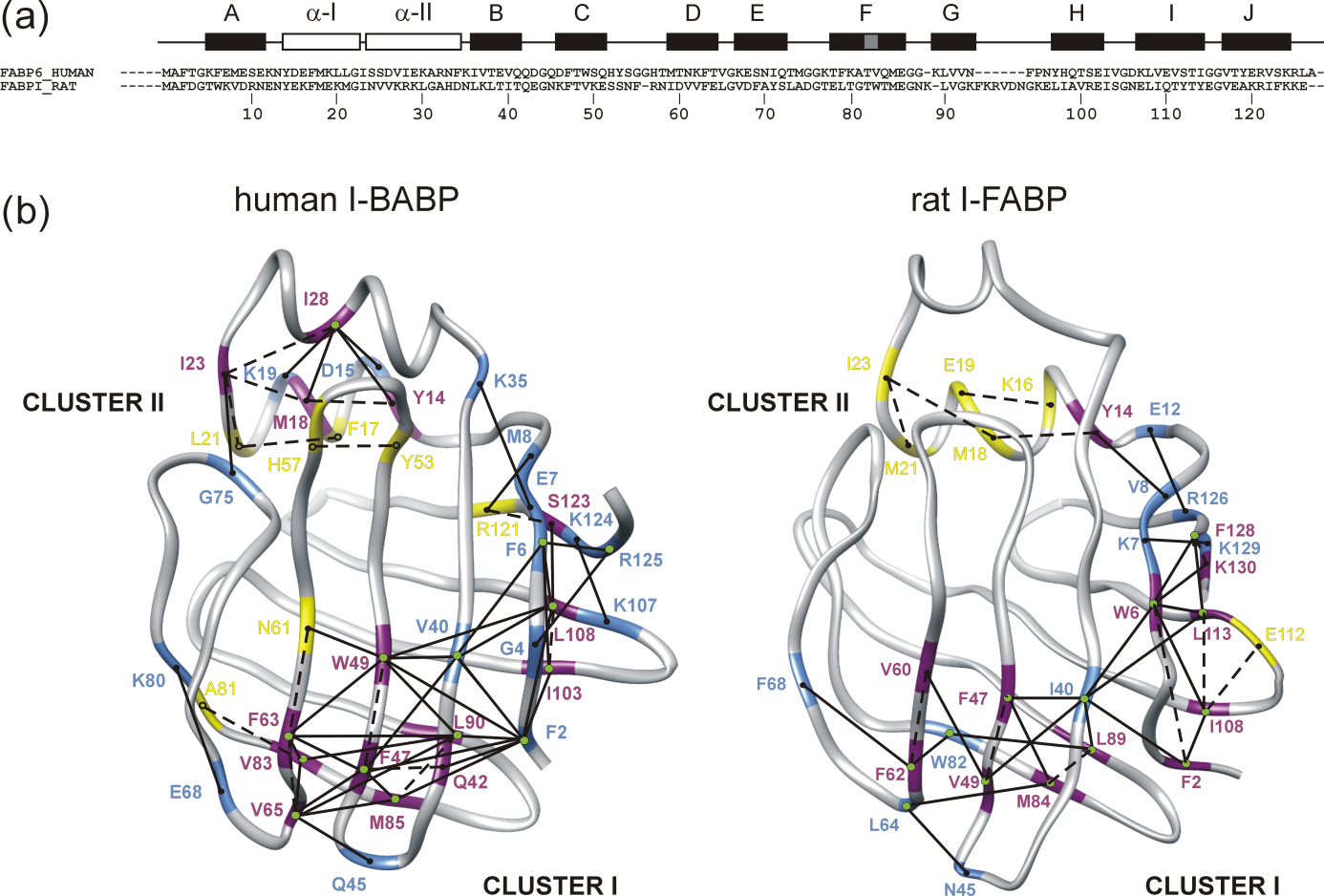
*

**Figure S3.** (a) Sequence alignment of human I-BABP and rat I-FABP discussed in the main text. Residue numbering is according to hI-BABP. Secondary structural elements indicated at the top are according to *apo* hI-BABP. The break occurring in βF is indicated in grey. (b) Conserved pairwise interactions in human I-BABP (PDB: 1O1U (Kurz et al., 2003), *left*) and rat I-FABP (PDB: 1AEL (Hodsdon and Cistola, 1997a), *right*). Short- and long-range interactions are depicted in dashed and solid lines, respectively. Residues involved in short-range, long-range, or both short- and long-range interactions are depicted in yellow, blue, and magenta, respectively. Residues involved in multiple long-range interactions are marked with a green dot. Note the missing residues in panel (A) affecting the numbering in panel (b).

| **Table S1.** ‘Local’ thermodynamic parameters of pressure-induced unfolding obtained for *apo* hI-BABP in 20 mM K-phosphate, 50 mM KCl, 0.05% NaN_3_, pH=6.3 at 291 K. | | |
| --- | --- | --- |
| Residue | ΔG° (kJ/mol) | ΔV° (mL/mol) |
| T3 | 12.0±0.6 | -54.1±2.7 |
| K5 | 3.4±0.7 | -24.8±4.1 |
| F6 | 12.3±0.9 | -57.8±4.1 |
| E7 | 3.4±0.6 | -24.8±3.7 |
| M8 | 14.0±1.0 | -64.5±4.4 |
| E9 | 14.2±0.8 | -59.8±3.3 |
| S10 | 17.2±1.1 | -71.1±4.6 |
| N13 | 14.2±1.0 | -62.4±4.6 |
| D15 | 17.4±0.9 | -71.7±3.8 |
| F17 | 16.7±0.8 | -72.5±3.6 |
| M18 | 16.5±0.8 | -72.4±3.5 |
| K19 | 16.8±0.8 | -68.3±3.4 |
| L20 | 17.8±0.9 | -69.7±3.4 |
| G22 | 16.8±1.1 | -70.0±4.7 |
| I23 | 16.4±0.8 | -72.1±3.3 |
| S24 | 16.7±1.1 | -70.4±4.5 |
| D26 | 14.3±1.1 | -62.8±5.1 |
| V27 | 17.1±0.8 | -70.2±3.3 |
| E29 | 18.2±0.9 | -65.5±3.1 |
| K30 | 14.3±1.1 | -63.8±4.8 |
| R32 | 14.3±0.9 | -61.6±3.7 |
| F34 | 14.3±0.8 | -63.5±3.3 |
| K35 | 17.7±0.9 | -72.5±3.6 |
| I36 | 18.4±0.8 | -66.0±4.0 |
| T38 | 14.1±1.0 | -63.7±4.7 |
| E39 | 16.8±1.0 | -75.1±4.5 |
| V40 | 17.0±1.2 | -67.7±5.5 |
| Q41 | 14.1±0.7 | -62.1±3.0 |
| Q42 | 16.8±0.9 | -70.4±3.7 |
| G44 | 17.0±0.5 | -71.4±4.5 |
| F47 | 14.2±0.9 | -61.1±4.2 |
| T48 | 14.1±0.6 | -63.5±2.9 |
| W49 | 14.3±1.3 | -58.7±5.7 |
| S54 | 16.8±1.1 | -69.7±4.7 |
| G55 | 17.5±1.0 | -65.9±4.1 |
| G56 | 11.9±0.8 | -54.1±3.8 |
| M59 | 16.3±1.0 | -66.5±4.3 |
| T60 | 10.2±1.0 | -45.9±4.6 |
| N61 | 16.6±0.9 | -65.6±3.8 |
| K62 | 12.0±0.8 | -49.5±3.3 |
| F63 | 16.4±0.8 | -75.8±3.9 |
| T64 | 17.5±0.7 | -68.0±2.9 |
| G66 | 17.5±0.9 | -65.4±3.7 |
| K67 | 17.7±0.7 | -62.7±4.2 |
| S69 | 17.9±1.1 | -68.1±4.5 |
| N70 | 16.3±0.7 | -72.2±3.3 |
| I71 | 11.9±0.8 | -56.3±3.7 |
| **Table S1.** Continuation. ‘Local’ thermodynamic parameters of pressure-induced unfolding obtained for *apo* hI-BABP in 20 mM K-phosphate, 50 mM KCl, 0.05% NaN_3_, pH=6.3 at 291 K. | | |
| Residue | ΔG° (kJ/mol) | ΔV° (mL/mol) |
| M74 | 17.1±0.7 | -72.8±4.9 |
| T78 | 18.3±1.1 | -68.5±4.7 |
| K80 | 17.3±0.9 | -72.4±5.1 |
| T82 | 14.1±0.9 | -62.6±3.9 |
| V83 | 11.9±1.0 | -54.5±4.8 |
| Q84 | 17.1±1.0 | -71.2±4.2 |
| M85 | 13.5±1.0 | -60.0±4.3 |
| L90 | 16.2±0.7 | -69.4±3.6 |
| N93 | 17.9±0.9 | -66.6±3.8 |
| T100 | 18.0±1.0 | -70.3±3.9 |
| V104 | 16.5±1.0 | -77.2±4.4 |
| G105 | 18.1±1.0 | -72.1±3.8 |
| K107 | 17.5±1.0 | -74.7±4.4 |
| V109 | 12.0±0.7 | -54.7±3.6 |
| G115 | 18.5±0.9 | -71.4±4.6 |
| G116 | 19.0±0.7 | -74.8±3.7 |
| V117 | 18.2±1.0 | -68.7±3.4 |
| Y119 | 10.2±0.5 | -48.6±2.2 |
| V122 | 10.2±0.6 | -50.3±3.2 |
| S123 | 10.2±0.6 | -42.0±3.5 |
| K124 | 12.1±0.9 | -48.7±4.1 |
| L126 | 12.1±0.7 | -52.4±3.1 |
| A127 | 14.5±1.0 | -61.1±4.1 |

| **Table S2.** Exchange parameters derived from global fit analysis of ^15^N relaxation dispersion curves assuming a two-state exchange obtained for *apo* hI-BABP in 20 mM K-phosphate, 50 mM KCl, 0.05% NaN_3_, pH=6.3 at 291 K, 1 bar. | | | | | | |
| --- | --- | --- | --- | --- | --- | --- |
| Residue | k_ex_ (s^-1^) | p_B_ (%) | Δω (Hz) | R_ex_ | R_2,eff_ | Φ (Hz^2^) |
| Group I | | | | | | |
| E68 | 1490 ± 135 | 4.4 ± 0.2 | 71±6 | 5.4±0.3 | 7.6±0.3 | 208±36 |
| N70 |  |  | 53±4 | 2.9±0.4 | 7.7±0.3 | 114±18 |
| I71 |  |  | 68±5 | 3.2±0.3 | 9.0±0.3 | 189±29 |
| Q72 |  |  | 51±4 | 2.6±0.3 | 11.2±0.3 | 105±17 |
| T73 |  |  | 165±10 | 17.7±0.4 | 11.6±0.3 | 1109±147 |
| M74 |  |  | 71±6 | 4.8±0.2 | 12.1±0.4 | 207±36 |
| T78 |  |  | 84±7 | 6.5±0.3 | 9.4±0.3 | 290±50 |
| A81 |  |  | 70±6 | 4.4±0.2 | 9.7±0.3 | 201±36 |
| T82 |  |  | 57±5 | 2.8±0.2 | 10.1±0.3 | 134±24 |
| G88 |  |  | 76±7 | 4.9±0.3 | 13.1±0.3 | 238±45 |
| V91 |  |  | 191±9 | 22.6±0.4 | 13.7±0.3 | 1484±164 |
| N93 |  |  | 61±5 | 3.1±0.2 | 11.2±0.4 | 151±26 |
| F94 |  |  | 45±4 | 2.3±0.2 | 8.9±0.3 | 84±15 |
| N96 |  |  | 127±9 | 12.3±0.3 | 16.5±0.5 | 657±99 |
| Y97 |  |  | 95±8 | 6.1±0.2 | 8.4±0.3 | 367±64 |
| H98 |  |  | 119±10 | 11.0±0.3 | 14.6±0.3 | 574±100 |
| Q99 |  |  | 92±8 | 5.6±0.2 | 10.1±0.3 | 348±62 |
| T100 |  |  | 87±8 | 4.7±0.3 | 11.1±0.4 | 306±58 |
| S101 |  |  | 64±5 | 4.2±0.2 | 11.1±0.3 | 167±27 |
| E102 |  |  | 53±4 | 2.7±0.3 | 10.7±0.3 | 118±19 |
| G105 |  |  | 41±4 | 2.6±0.2 | 10.2±0.3 | 69±14 |
| V109 |  |  | 50±5 | 2.9±0.3 | 10.0±0.4 | 102±21 |
| E110 |  |  | 58±6 | 3.1±0.2 | 10.7±0.3 | 139±29 |
| T113 |  |  | 45±4 | 1.8±0.2 | 10.8±0.3 | 83±15 |
| G115 |  |  | 52±5 | 2.1±0.2 | 8.9±0.3 | 108±21 |
| Y119 |  |  | 40±4 | 2.6±0.2 | 11.8±0.4 | 67±13 |
| V122 |  |  | 55±4 | 3.0±0.3 | 10.0±0.3 | 125±19 |
| Group II | | | | | | |
| E7 | 862 ± 102 | 2.2 ± 0.2 | 43±4 | 2.0±0.2 | 10.5±0.3 | 40±8 |
| E11 |  |  | 55±5 | 2.4±0.3 | 8.7±0.2 | 64±12 |
| N13 |  |  | 59±4 | 2.7±0.4 | 10.0±0.3 | 74±11 |
| F17 |  |  | 64±5 | 3.1±0.4 | 11.5±0.4 | 87±14 |
| K30 |  |  | 41±5 | 1.5±0.3 | 10.6±0.3 | 36±9 |
| K35 |  |  | 68±7 | 3.4±0.4 | 14.3±0.4 | 96±20 |
| E39 |  |  | 63±5 | 3.5±0.5 | 12.2±0.3 | 84±13 |
| V40 |  |  | 59±5 | 2.7±0.2 | 11.7±0.4 | 72±12 |
| H52 |  |  | 33±4 | 1.1±0.2 | 10.9±0.2 | 23±6 |
| G55 |  |  | 73±8 | 3.9±0.4 | 12.1±0.4 | 113±25 |
| H57 |  |  | 63±5 | 3.1±0.3 | 7.3±0.2 | 85±14 |
| T58 |  |  | 64±6 | 3.1±0.3 | 9.8±0.3 | 87±17 |
| T60 |  |  | 66±4 | 3.3±0.3 | 10.4±0.3 | 92±12 |
| N61 |  |  | 65±3 | 3.2±0.3 | 11.0±0.3 | 89±10 |
| K62 |  |  | 62±4 | 2.9±0.3 | 10.0±0.2 | 80±11 |
| F63 |  |  | 46±4 | 1.8±0.2 | 10.2±0.3 | 45±8 |
| **Table S3.** Exchange parameters derived from global fit analysis of ^15^N relaxation dispersion curves assuming a two-state exchange obtained for *apo* hI-BABP in 20 mM K-phosphate, 50 mM KCl, 0.05% NaN_3_, pH=6.3 at 291 K, 2000 bar. | | | | | | |
| Residue | k_ex_ (s^-1^) | p_B_ (%) | Δω (Hz) | R_ex_ | R_2,eff_ | Φ (Hz^2^) |
| F2 | 427 ± 71 | 3.8 ± 0.2 | 62±4 | 6.9±0.6 | 19.5±0.3 | 138±19 |
| T3 |  |  | 33±6 | 2.7±0.8 | 11.3±0.3 | 38±14 |
| A31 |  |  | 57±5 | 6.3±0.7 | 13.1±0.3 | 117±21 |
| R32 |  |  | 44±5 | 4.3±0.7 | 13.1±0.3 | 69±15 |
| I36 |  |  | 49±6 | 5.1±0.8 | 16.4±0.3 | 87±19 |
| V37 |  |  | 48±6 | 5.0±0.8 | 12.0±0.3 | 83±19 |
| T38 |  |  | 54±5 | 5.8±0.7 | 13.9±0.3 | 105±19 |
| E39 |  |  | 56±5 | 6.1±0.7 | 13.7±0.3 | 113±21 |
| Q45 |  |  | 88±5 | 9.9±0.5 | 13.2±0.3 | 281±29 |
| F47 |  |  | 42±6 | 4.1±0.8 | 11.4±0.3 | 64±17 |
| N61 |  |  | 60±6 | 6.7±0.8 | 14.9±0.3 | 128±25 |
| K62 |  |  | 113±5 | 11.7±0.5 | 10.3±0.3 | 465±46 |
| F63 |  |  | 92±5 | 10.2±0.5 | 10.7±0.3 | 304±33 |
| I71 |  |  | 38±6 | 3.5±0.8 | 11.0±0.3 | 53±16 |
| Q72 |  |  | 78±5 | 8.9±0.5 | 13.5±0.3 | 219±27 |
| T73 |  |  | 134±3 | 12.7±0.5 | 13.3±0.3 | 652±40 |
| K77 |  |  | 49±6 | 5.1±0.8 | 11.4±0.3 | 86±19 |
| T78 |  |  | 46±5 | 4.6±0.8 | 11.8±0.3 | 75±16 |
| T82 |  |  | 72±6 | 8.2±0.7 | 10.2±0.3 | 188±30 |
| L90 |  |  | 82±4 | 9.3±0.5 | 15.2±0.3 | 243±25 |
| V92 |  |  | 53±6 | 5.8±0.9 | 12.4±0.3 | 102±23 |
| H98 |  |  | 43±5 | 4.3±0.7 | 13.7±0.3 | 67±14 |
| Q99 |  |  | 35±5 | 3.0±0.7 | 13.2±0.3 | 44±13 |
| V109 |  |  | 47±6 | 4.8±0.8 | 12.2±0.3 | 79±18 |
| T113 |  |  | 25±6 | 1.7±0.7 | 13.9±0.3 | 22±11 |
| T118 |  |  | 66±5 | 7.5±0.7 | 11.9±0.3 | 157±25 |
| V119 |  |  | 66±5 | 7.6±0.7 | 10.7±0.3 | 160±26 |
| R121 |  |  | 63±6 | 7.2±0.8 | 11.6±0.3 | 146±28 |
| V122 |  |  | 46±5 | 4.7±0.7 | 13.3±0.3 | 77±17 |
